# Tailoring Alkyl Side Chains of Ionizable Amino-Polyesters for Enhanced In Vivo mRNA Delivery

**DOI:** 10.1021/acsabm.5c00116

**Published:** 2025-04-28

**Authors:** Aida López Espinar, Lianne M. Mulder, Mohamed Elkhashab, Zahra Khan, Mariusz Czarnocki-Cieciura, Maria R. Aburto, Sonja Vucen, Piotr S. Kowalski

**Affiliations:** † School of Pharmacy, University College Cork, Cork T12 K8AF, Ireland; ‡ Laboratory of Protein Structure, International Institute of Molecular and Cell Biology, Warsaw 02-109, Poland; § APC Microbiome Ireland, University College Cork, Cork T12 K8AF, Ireland; ∥ Department of Anatomy and Neuroscience, University College Cork, Cork T12 K8AF, Ireland; ⊥ SSPC, Research Ireland Centre for Pharmaceuticals, School of Pharmacy, University College Cork, Cork T12 K8AF, Ireland

**Keywords:** nucleic acid delivery, mRNA, polymeric nanoparticles, polyesters, biomaterials

## Abstract

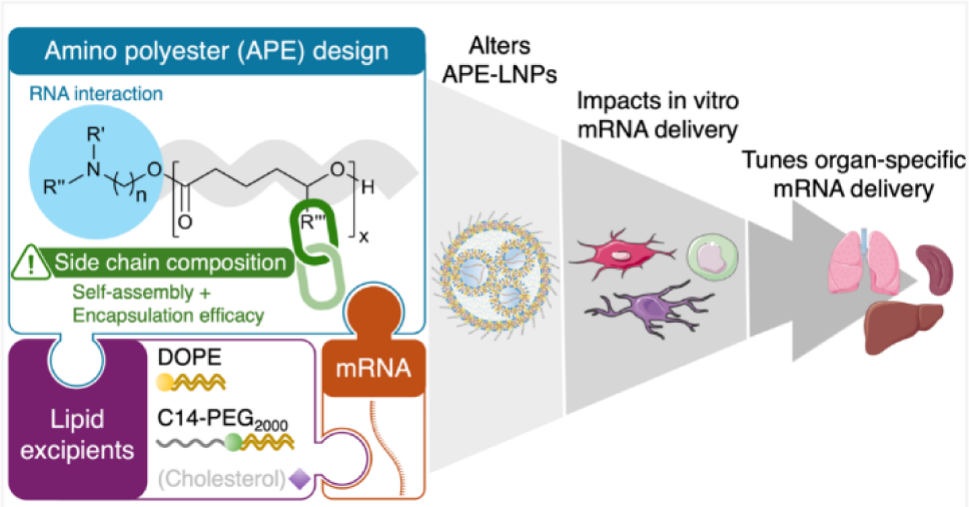

Lipid nanoparticles (LNPs) containing ionizable lipids are the most clinically advanced platform for mRNA delivery, but their application beyond the liver remains challenging. Polymer–lipid hybrid nanoparticles offer a promising alternative, combining the synthetic versatility and unique properties of polymers with the biocompatibility of lipid excipients. While the significance of alkyl tail design is well-recognized for ionizable lipids, the impact of the polymer side chain composition on interactions with lipid excipients, mRNA delivery efficacy, and tissue specificity remains poorly understood. Here, we focus on a class of ionizable amino-polyesters (APEs) that exhibit features desired for potential clinical applications, including narrow molecular weight distribution and a good safety profile, and investigate the effect of polymer side chain composition on the formulation of APE lipid nanoparticles (APE-LNPs) for mRNA delivery. A library of 36 APEs was synthesized via ring-opening polymerization of chemically diverse tertiary amino-alcohols and lactone monomers with distinct alkyl side chain compositions, including variations in length and unsaturation. We show that optimal alkyl side chain length is critical for the assembly of stable mRNA nanoparticles and efficient mRNA delivery both *in vitro* and *in vivo*. Top-performing APE-LNPs display superior delivery efficacy *in vitro* and in extrahepatic tissues compared to benchmark LNPs, including DLin-MC3-DMA ionizable lipid. The polymer chain composition affects the tissue selectivity of APE-LNPs, with shorter side chains (4–5 carbons) effectively targeting the spleen and lungs, while longer chains (7–9 carbons) show enhanced liver delivery. We also explored the relevance of lipid excipients in APE-LNPs, demonstrating the essential role of unsaturated phospholipids in enhancing cellular uptake and mRNA delivery, and the limited relevance of cholesterol. These findings provide valuable insights into the design of polymers for use in the LNP context, which could aid the development of polymeric alternatives to ionizable lipids and expand the utility of mRNA LNP technology to nonliver tissues.

## Introduction

The messenger RNA (mRNA) technology has been recognized for its global success as a vaccine against COVID-19 and its prospects to revolutionize gene editing and cancer treatment.^1−5^ Lipid nanoparticles (LNPs) containing ionizable cationic lipids have emerged as the most clinically advanced platform for mRNA delivery, but their application for delivery to nonliver tissues faces challenges related to preferential uptake by liver hepatocytes, which can be traced even after intramuscular administration.^6−9^ LNP formulations, including charged lipids, enable targeting to the lungs or spleen,^10,11^ however, the risk of thrombosis caused by the cationic component has been implicated as a major barrier for safe systemic mRNA delivery to the lungs.^12^ Recent studies also link endosomal escape, facilitated by ionizable and cationic lipids, to inflammatory responses that can exacerbate preexisting inflammation and should be taken into consideration for the treatment of diseases with underlying inflammatory conditions.^13^ Exploring alternatives to ionizable cationic lipids is, therefore, paramount to complementing the strengths of LNPs and helping expand the therapeutic utility of mRNA.

Polymers have been widely utilized in drug delivery. They are chemically versatile and possess unique physical and mechanical properties that can be leveraged to improve mRNA delivery to extrahepatic tissues and obtain materials with increased biocompatibility and tissue selectivity.^14−17^ The application of controlled polymerization methods, such as ring opening polymerization (ROP) and radical polymerization yields more defined polymers, significantly increasing their potential for clinical translation and positioning them as a promising alternative to lipids for the development of mRNA delivery systems.^17−20^ Polyesters are among the most promising synthetic polymers for mRNA delivery. They degrade in a biological milieu via hydrolysis of ester linkages and show good safety profiles *in vivo*.^21^ To enable mRNA encapsulation, various types of cationic polyesters have been developed, including poly(β-amino esters) (PBAEs), poly(amino coesters) (PACEs), and charge-altering releasable transporters (CARTs), which have shown efficient mRNA delivery in different tissues.^22−24^ Recently, Kowalski et al. reported a new class of ionizable amino polyesters (APEs) synthesized via ROP of lactones and tertiary amino-alcohols that display narrow molecular weight distribution and good batch-to-batch consistency, which are desired for clinical translation.^25^ The ionizable behavior of the tertiary amines and the incorporation of lactone monomers, which are recognized as safe by the Food and Drug Administration (FDA), contribute to their biocompatibility.^26^ While polyesters used for mRNA delivery are directly complexed with the nucleic acid, forming a polyplex or coformulated with polyethylene glycol (PEG) or poloxamers, APEs can be incorporated into lipid nanoparticles (APE-LNPs) with various excipients such as cholesterol, phospholipids, and PEG-lipids, presenting a potential alternative to ionizable lipids.^25,27,28^

Previous studies have established that the chemical composition of APEs, in particular the presence of an alkyl side chain in the polymer, is one of the key factors contributing to efficient mRNA delivery to various tissues.^25,29^ Similarly, the alkyl tail composition, including unsaturation and branching, was reported to have a significant contribution to improving the design of ionizable lipids for mRNA delivery.^30,31^ Despite the recognized significance of alkyl tail composition for LNP engineering, there is currently limited understanding of how polymer side chain composition can influence the interaction with lipid excipients, mRNA delivery efficacy, and tissue specificity. Closing this knowledge gap could inform the design of polymers for use in the context of LNPs and help extend the applicability of LNP technology to nonliver tissues.

In this study, we investigate the effect of APE side chain composition on the design of APE-LNPs for mRNA delivery to better understand the potential of APEs as an alternative for ionizable lipids in LNP formulation. We synthesized a library of 36 APEs composed of six chemically diverse tertiary amino alcohols and lactone monomers with different side chain compositions and formulated them into APE-LNPs. We evaluated the impact of the side chain composition on the physicochemical properties of both the polymer and APE-LNPs, and their mRNA delivery efficacy *in vitro* and *in vivo*, supporting the significance of the polymer side chain design. We also studied the relevance of the helper lipids in the APE-LNP formulation, revealing their role in enhancing the mRNA delivery efficacy.

## Results and Discussion

### Design and Characterization of the APE Library

**Figure 1:**
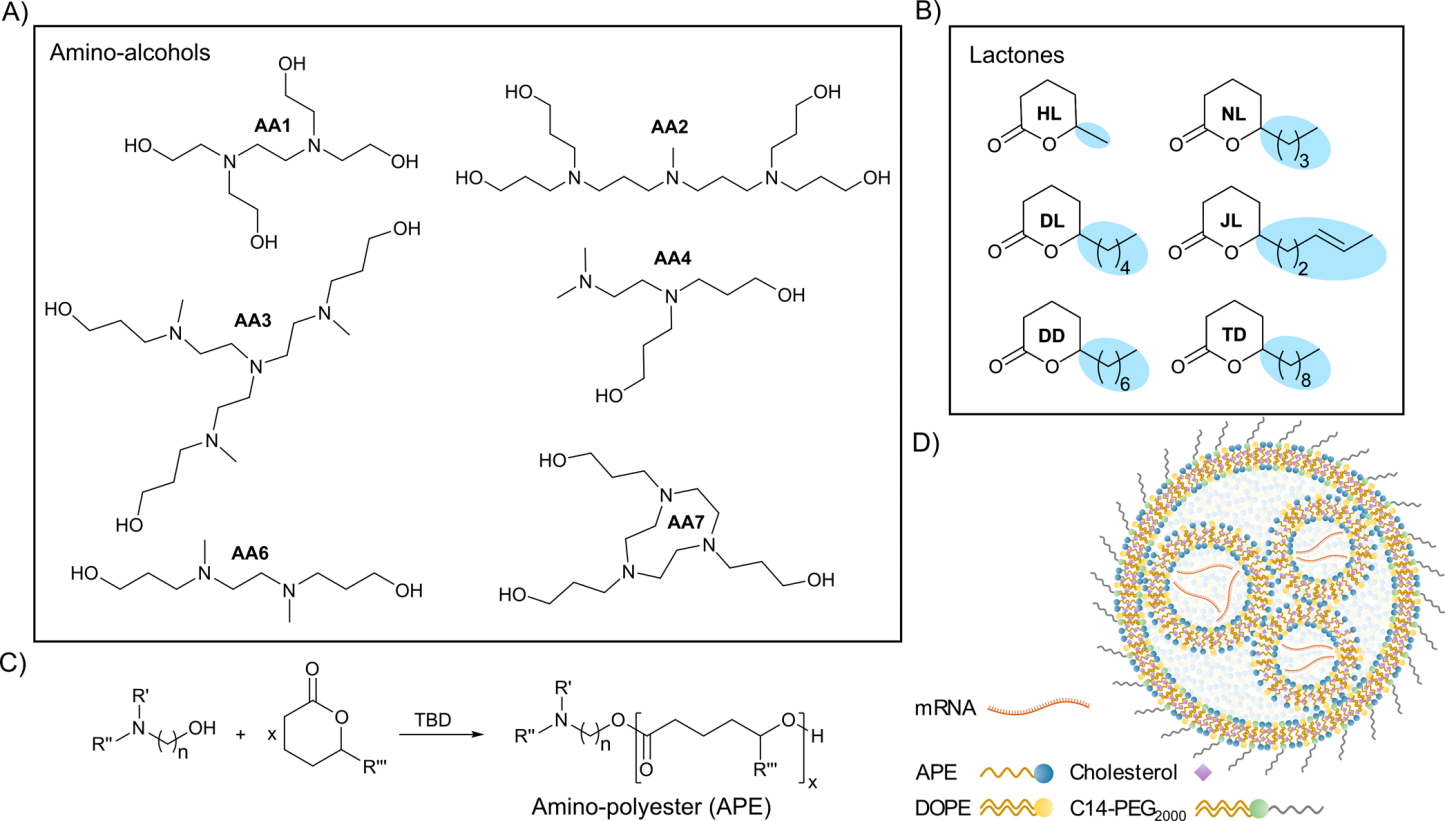
Components used for the synthesis of the APEs library: (A) amino alcohols (AA1–AA7) and (B) lactones [δ-hexanolactone (HL), δ-nonalactone (NL), 5-decanolide (DL), jasmolactone (JL), δ-dodecalactone (DD) and δ-tetradecalactone (TD)]. The side chain composition of the lactones is highlighted in blue. (C) Schematic of the APE synthesis via ROP. (D) Representation of the APE-LNP composition.

Our previous work demonstrated that the presence of the alkyl side chain in the lactone monomer was critical for effective mRNA delivery by APE-LNPs.^25,29^ Here, we aimed to better understand the effect of the alkyl side chain composition of the APE on its physical and thermal properties, as well as its ability to assemble into lipid nanoparticles and deliver mRNA. To this end, a library of 36 APEs was synthesized using six chemically diverse tertiary amino-alcohols (AA) (Figure 1A) and lactones [δ-hexanolactone (HL), δ-nonalactone (NL), 5-decanolide (DL), jasmolactone (JL), δ-dodecalactone (DD), and δ-tetradecalactone (TD)] with varying alkyl side chain lengths (ranging from one to nine carbons, C1–C9) and compositions (including the presence of unsaturated bonds) (Figure 1B). Six-membered ring lactones were selected to ensure that polymer composition was not impacted by the dimension of the lactone ring. Jasmolactone (JL) was included to study the effect of the unsaturated bond in the alkyl side chain, as unsaturated alkyl tails have been shown to enhance mRNA delivery of ionizable lipids.^31,32^ We previously observed that using amino-alcohols with a higher number of tertiary amines improved APE mRNA delivery efficacy;^25^ therefore, linear and branched amino-alcohols (AA2, AA4, AA3, AA6, AA7) with two to four tertiary amines and hydroxyl groups were designed and synthesized for this study. APEs were prepared via ROP of the lactone, initiated by the tertiary AA in the presence of triazabicyclodecene (TBD) as a catalyst (Figure 1C) as described in the Materials and methods section. The degree of polymerization was set to three monomer repeat units per hydroxyl group of the initiator, which had previously yielded APEs with the highest mRNA delivery efficacy.^25^

All APEs were characterized before and after purification by gel permeation chromatography (GPC) and NMR showing narrow molecular weight distribution (Đ 1.23–1.46), high monomer conversion (>80%), and degree of polymerization (q) close to the theoretical as determined by ^1^H NMR (Figure S1 and Table S1). The high efficacy of the ROP and good control of the polymer molecular weight (Mn) (Figure S2) featured by the APE platform help facilitate the systematic assessment of the polymer structure–activity relationship (SAR) for mRNA delivery. Moreover, all APEs displayed low batch-to-batch variability for both the polymer properties and their corresponding LNPs (Tables 1 and S2), which are relevant to addressing hurdles to the translation of polymeric nanomedicines.^17^

**Table 1:** Characterization of Selected APEs and Their APE-LNPs^e^

APE	Mn^a^^a^ [kDa]	Đ^a^ [−]	Diameter^b^ [nm]	PDI^b^^b^ [−]	ζ^b^ [mV]	mRNA EE^c^^c^ [%]	p*K*_a_^d^^d^
AA2-HL-3	1.69 ± 0.03	1.26 ± 0.02	338 ± 74	0.380 ± 0.16	–17.1 ± 2.5	56.9 ± 6.2	5.0 ± 0.1
AA2-NL-3	2.18 ± 0.22	1.27 ± 0.06	90 ± 6	0.091 ± 0.03	–5.3 ± 5.6	85.0 ± 3.8	4.8 ± 0
AA2-DL-3	2.26 ± 0.34	1.25 ± 0.05	96 ± 13	0.161 ± 0.01	–4.3 ± 2.9	92.3 ± 4.5	4.7 ± 0.3
AA2-JL-3	1.86 ± 0.14	1.29 ± 0.04	151 ± 10	0.130 ± 0.07	–10.3 ± 6.8	68.5 ± 14.0	4.3 ± 0
AA2-DD-3	3.12 ± 0.34	1.22 ± 0.02	75 ± 0	0.154 ± 0.01	–4.5 ± 3.1	93.3 ± 2.3	4.7 ± 0.1
AA2-TD-3	4.70 ± 0.54	1.21 ± 0.00	77 ± 5	0.173 ± 0.04	–8.7 ± 10.2	94.6 ± 1.6	4.3 ± 0.4

a Characterized by gel-permeation chromatography.

b Characterized by dynamic light scattering.

c Characterized by RiboGreen.

d Characterized by TNS assay; Mn: number-average molecular weight, Đ: dispersity, PDI: polydispersity index, ζ: zeta potential, EE: encapsulation efficacy.

e Data are represented as mean ± SD from two independently synthesized batches of each polymer and APE–LNPs (n = 2).

**Figure 2:**
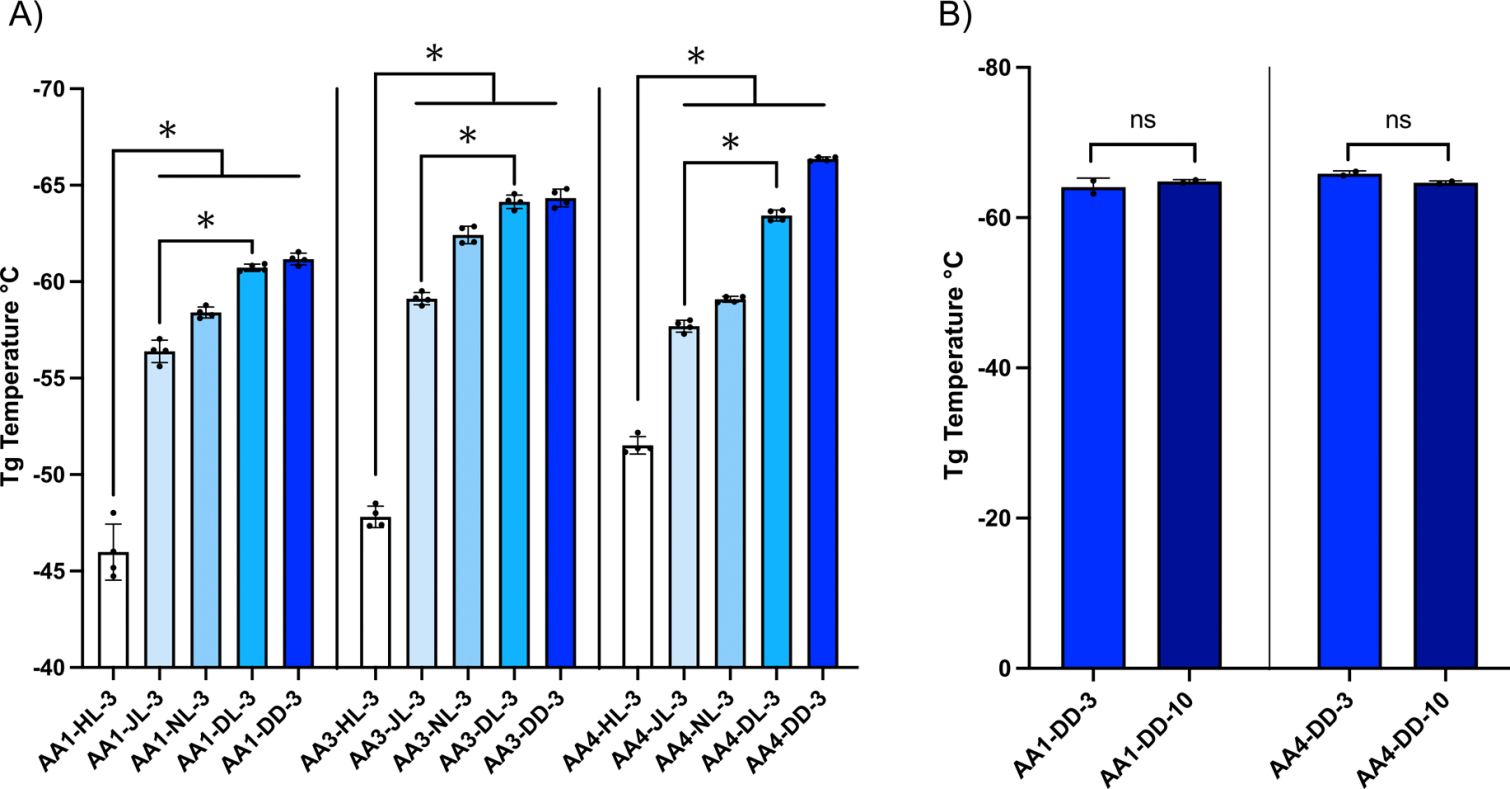
Glass transition temperature (Tg) analysis. (A) Tg of polymers composed of amino-alcohols AA1, AA3, and AA4 and 3 repeat units of the lactone with different lengths of the alkyl side chain (from shortest to longest; HL < NL < JL = DL < DD); * *p* < 0.0001. (B) Tg for AA1-DD and AA4-DD with the degree of polymerization corresponding to 3 and 10 monomer repeat units, not significant (ns); *p* > 0.05.

As thermal properties are strongly linked to the polymer’s chemical and physical properties, we studied the impact of alkyl side chain composition on the glass transition temperature (Tg) of selected APEs. Tg is defined as the temperature at which a polymer transitions from a glassy state to a rubbery state. This transition is influenced by structural factors that prevent alignment among the macromolecules and promote the mobility of the polymer chains, e.g., pendant side groups generally lead to a lower Tg.^33^ Hence, it is expected that longer side chains with higher rotational freedom would decrease the Tg of the APE, while the presence of unsaturated bonds or bulky groups would introduce rigidity to the side chain, resulting in an increased Tg of the polymer.^34,35^ All studied APEs were amorphous (Figure S3) with Tg ranging from −45°C to −65°C (Figure 2A). We found that the Tg of the polymers was primarily affected by the composition of the side chain rather than the amino alcohol. Increasing the length of the alkyl side chain corresponded to significantly lower Tg (Figure 2A). The incorporation of an unsaturated bond in the polymer side chain (AA1-JL-3, AA3-JL-3, and AA4-JL-3) significantly increased the Tg compared with APEs with corresponding saturated side chains (AA1-DL-3, AA3-DL-3, and AA4-DL-3), likely due to the higher rigidity of the double bond. We also confirmed that the MW of APE did not impact Tg by comparing polymers synthesized with three (AA1-DD-3 and AA3-DD-3) and ten monomer repeat units (AA1-DD-10 and AA3-DD-10, Figure 2B and Table S3). Our data show that the thermal properties of APEs are strongly linked to their side chain composition, and Tg analysis via differential scanning calorimetry can be used as a reliable method to complement the evaluation of APE composition and batch-to-batch consistency, enabling robust characterization of these polymers.

### The APE Side Chain Composition Impacts the Physicochemical Properties of APE-LNPs

Polymer–lipid hybrid nanoparticles present a promising strategy for mRNA delivery, allowing the integration of the synthetic versatility and unique properties of polymers with the modularity and biocompatibility of lipid excipients.^36,37^ Our previous work and others demonstrated that APEs synthesized with ε-caprolactone (CL, no side chain) had an impaired ability to form stable lipid nanoparticles with mRNA, regardless of the polymer MW, and required the presence of a side chain, suggesting that the composition of the alkyl side chain could play an important role in APEs’ interaction with lipid excipients.^25,29^ To better understand the impact of APE side chain composition on facilitating assembly into LNPs, we systematically investigated the physicochemical properties and mRNA delivery capacity of 36 APE-LNPs. The library of APEs was formulated into nanoparticles containing mRNA encoding firefly luciferase (FLuc), along with 1,2-dioleoyl-*sn*-glycero-3-phosphoethanolamine (DOPE), cholesterol, and 1,2-dimyristoyl-*sn*-glycero-3-phosphoethanolamine-*N*-[methoxy-(polyethylene glycol)-2000] (ammonium salt) (C14-PEG_2000_) using microfluidic mixing. Nanoparticles were prepared at a 9:1 ratio of tertiary amines in the amino polyester to mRNA phosphate groups (N/P; Figure 1D).

**Figure 3:**
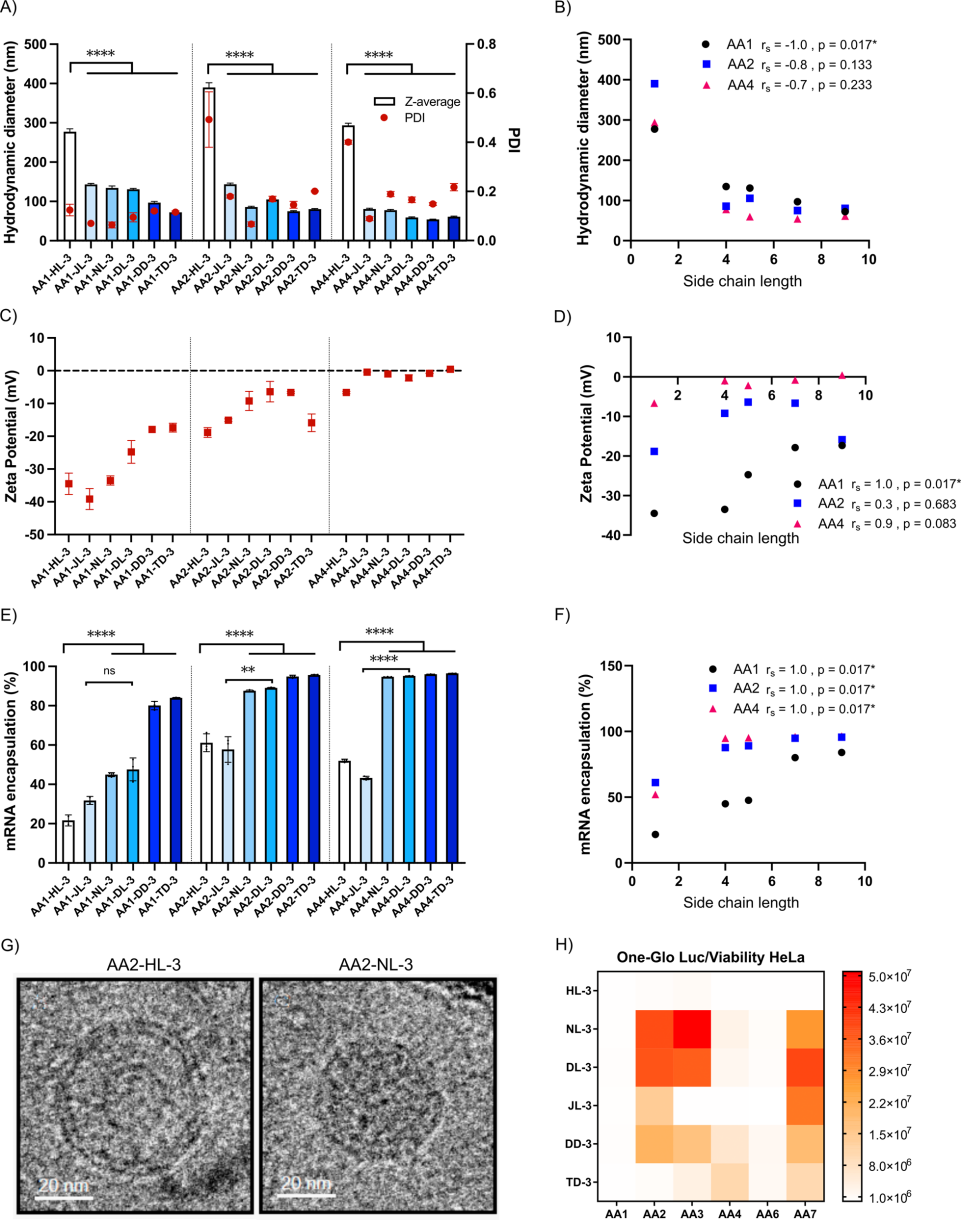
Characterization of physicochemical properties and *in vitro* mRNA delivery capabilities of APE-LNPs. (A) Hydrodynamic diameter (nm) and polydispersity index (PDI) of APE-LNPs formulated with polymers AA1, AA2, and AA4 with varying lengths of the alkyl side chain. Data are presented as mean ± SD; *n* = 3; ****p < 0.000 1. (C) Surface charge of APE-LNPs. Data are presented as mean ± SD; *n* = 2. (E) mRNA encapsulation efficacy of APE-LNPs measured by Ribogreen assay. Data are presented as mean ± SD; *n* = 3; ns, *p* > 0.05, *p < 0.05, **p < 0.001, ****p < 0.0001. Spearman correlation coefficient between polymer alkyl side chain length and (B) hydrodynamic diameter, (D) surface charge, and (F) mRNA encapsulation of APE-LNPs. (G) Representative Cryo-TEM images of AA2-HL-3 and AA2-NL-3 nanoparticles. The scale bar represents 20 nm. (H) *In vitro* evaluation of mRNA delivery efficacy by APE-LNPs containing FLuc mRNA in HeLa cells. Cells were transfected for 24 h with 50 ng of FLuc mRNA. Data are presented as mean relative luminescence (normalized to viability); *n* = 3.

We found that the APE alkyl side chain length had a significant impact on the APE-LNPs’ physicochemical properties, including their hydrodynamic diameter, surface charge, and mRNA encapsulation efficacy. In particular, APE-LNPs formulated with HL polymers containing single-carbon (C1) side chains were significantly larger and more polydisperse (hydrodynamic diameters ranging from 150 to 400 nm and PDIs from 0.2 to 0.6) compared to APE-LNPs formulated with polymers containing side chains between four and nine carbons (C4–C9). The latter yielded homogeneous nanoparticles ranging from 65 to 140 nm in diameter and PDIs below 0.2 (Figures 3Aand S4A). Cryogenic electron microscopy (cryo-TEM) also confirmed the different morphology of the APE-LNPs composed of HL polymer, indicative of impaired nanoparticle assembly and poor mRNA encapsulation (presence of multi- and unilamellar vesicles with low electron density; Figures 3G and S5). Overall, a strong negative correlation between alkyl side chain length and the size of APE-LNPs was observed for all polymers, demonstrating the critical importance of the APE side chain design for their ability to form nanoparticles with lipid excipients (Figures 3B and S4B).

Moreover, the APE-LNPs composed of HL polymers (AA1-HL-3 and AA4-HL-3) showed limited stability over time compared to DL-based polymers (AA1-DL-3 and AA4-DL-3), increasing in size and polydispersity when stored in PBS (pH 7.4) at 4 °C for up to 28 days (Figure S6). This data confirm that the APE alkyl side chain length also plays a role in LNP stability, and polymers with short (e.g., C1) or no side chains display limited size stability or tend to form aggregates.^25,29^

The surface charge (ζ) of APE-LNPs ranged from −20 to 10 mV (Figures 3C and S4C ), and a positive correlation was observed between the polymer alkyl side chain length and the ζ-potential (Figures 3D and S4D). The mRNA encapsulation efficiency (EE) of the APE-LNPs was between 20 and 95% and also positively correlated with the alkyl side chain length. We hypothesize that APE-LNPs with low EE (<60%) may exhibit a decreased surface charge due to potential interference of the nonencapsulated mRNA. Polymers with side chains containing four or more carbons yielded particles with significantly increased EE (Figures 3E,F and S4E,F). We also observed that the presence of an unsaturated bond in the APE alkyl side chain (JL) significantly decreased the mRNA EE and ζ-potential for the majority of the APE-LNPs compared to its saturated counterpart (DL) but had a modest impact on the particle size in contrast to HL polymers (Figures 3A,C,E and S4A,C,E,). Liu etal. reported that cis double bonds introduced into polyesters functionalized with fatty acid chains impeded the packing of side chains and polymer, such behavior could potentially contribute to the lower mRNA EE of JL APE-LNPs but trans double bonds may not display the same behavior.^38^

### Polymer Alkyl Side Chain Length Impacts the mRNA Delivery Efficacy of APE-LNPs In Vitro

To establish the impact of the APE side chain composition on mRNA delivery efficacy, the library of APE-LNPs was formulated with Firefly Luciferase (Fluc) mRNA and evaluated in three relevant cell types, including HeLa cells, human leukemia monocytic cells (THP-1), and human umbilical vein endothelial cells (HUVECs). We found that the length of the polymer alkyl side chain played a major role in the efficacy of mRNA delivery by the APE-LNPs. APEs with C4 and C5 side chains (NL and DL) exhibited the highest efficacy, whereas polymers with a C1 side chain (HL) were unable to deliver the mRNA (Figures 3H and S7A,B), likely due to impaired interaction of the polymer with lipid excipients, resulting in low EE and large particles with limited stability (Tables 1 and S2). Similarly, the presence of an unsaturated bond in the polymer side chain (JL) impaired the mRNA delivery efficacy of most APE-LNPs compared to its saturated counterpart (DL) (Figures 3H and S7A,B). The lower performance of the JL polymers could be linked to impaired mRNA encapsulation; however, Fenton et al., showed that the placement, cis/trans geometry, and the optimal number of alkenes per tail were also critical for efficient mRNA delivery with ionizable lipids.^39^ Generally, APE-LNPs containing polymers with 3 or 4 tertiary amines showed better mRNA delivery efficacy compared to APE-LNPs containing 2 tertiary amines corroborating our previous results.^25^ Top-performing APE-LNPs containing AA2 and AA3 polymers with C4 and C5 side chains (NL, DL) showed up to 10-fold higher transfection efficacy in HeLa cells than polymers containing longer tails C7 and C9 (DD and TD) selected from the previous study (Figure 3H).^25^ Compared to benchmark LNPs, including C12–200 and MC3 ionizable cationic lipids, the AA2-LNPs showed up to 6- and 400-fold more efficient mRNA delivery in HeLa cells, respectively (Figure S7C). The majority of APE-LNPs were well tolerated in all the cell lines, with notable toxicity for polymers containing 4 tertiary amines (AA3) compared to control LNPs (Figure S8A–C). Interestingly, the mRNA delivery efficacy of some APE-LNPs was found to be cell-type dependent, with AA7-NL-3 and AA7-DL-3 only showing efficacy in HeLa cells (Figures 3H and S7A,B). These data show that the optimal side chain design is required for each polymer to maximize the potency of mRNA delivery to different cells by APE-LNPs.^40^ Branching of ionizable lipids tails was found to impact the volumetric ratio occupied by the headgroup and alky tails influencing the transition to inverted hexagonal phase H_II_ and facilitating ionization at pH 5, leading to enhanced endosomal escape.^41,42^ The best-performing APE-LNPs showed higher ionization scores at pH 5 than the HL and JL APE-LNPs (Figure S9C,D); hence, we hypothesize that side chain design may help facilitate better endosomal escape and consequently enhanced mRNA delivery. The APE side chain could also potentially contribute to improved endosomal escape through balancing the hydrophobicity of the polymer or changing its degradation profile by controlling access of water to an ester bond.^18,43,44^

### Efficient mRNA Delivery of APE-LNPs Requires the Incorporation of Unsaturated Phospholipids

**Figure 4:**
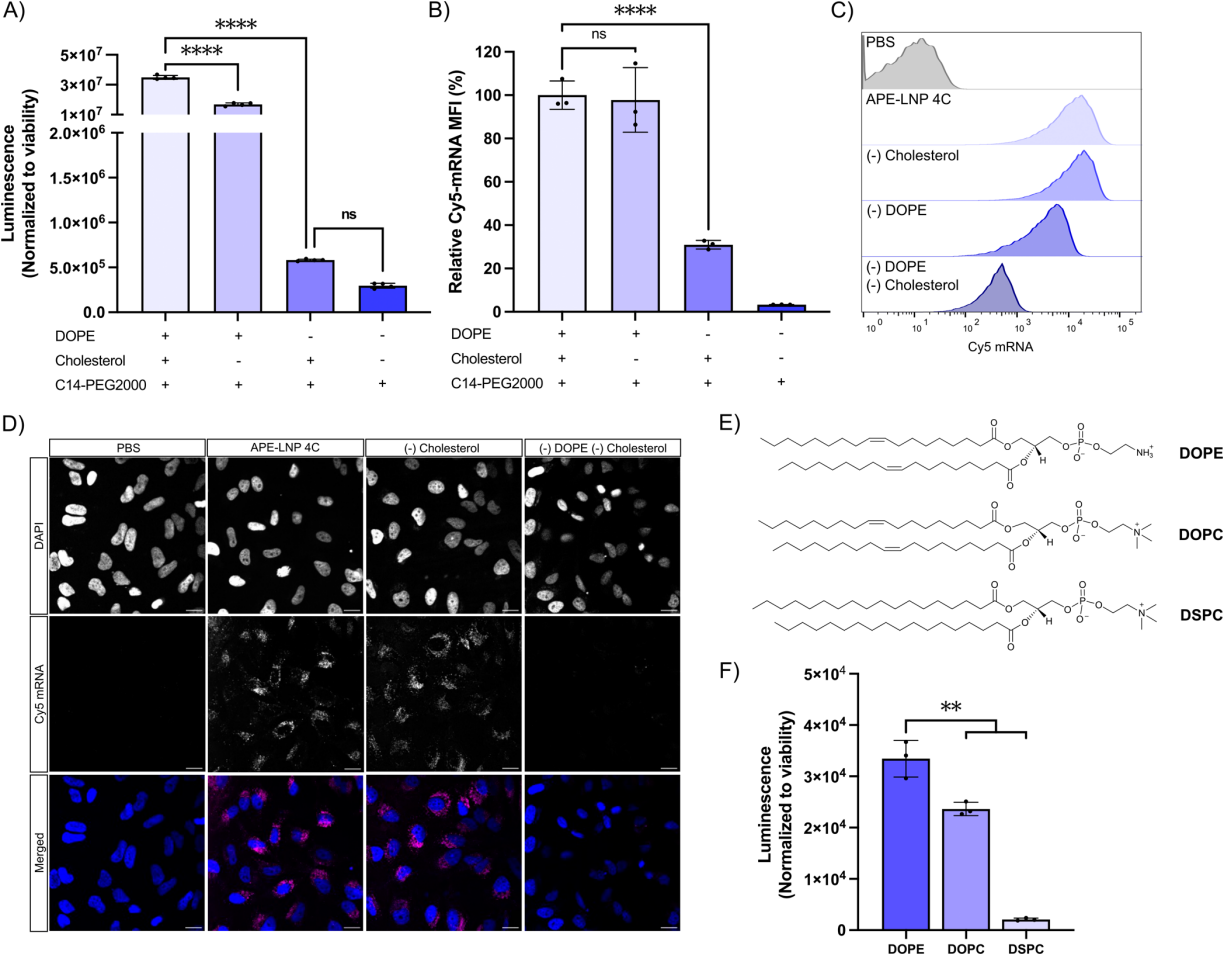
Investigation of the role of helper lipids in the APE-LNPs formulation. Hela cells transfected with AA2-NL-3 APE-LNPs with or without DOPE and/or cholesterol containing Cy5-labeled FLuc mRNA. (A) Normalized luminescence signal measured 24 h after transfection with 50 ng of FLuc mRNA. Data are presented as mean relative luminescence ± SD; *n* = 3. (B) Flow cytometry analysis of the uptake of 250 ng APE-LNPs containing Cy5 mRNA, 24 h after transfection. Mean fluorescence intensities of Cy5 are presented relative to 4-component APE-LNP (4C) as mean ± SD; *n* = 3. (C) Representative histograms of Cy5+ cell population. (D) Representative images of the uptake of APE-LNPs into HeLa cells with DAPI (blue) and Cy5 mRNA (magenta). Scale bars represent 20 μm. ****p < 0.0001. (E) Chemical structure of phospholipids DOPE, DOPC, and DSPC. (F) mRNA delivery efficacy of APE-LNPs incorporating these different phospholipids. Normalized luminescence signal measured 24 h after transfection of HeLa cells with APE-LNPs containing 50 ng of FLuc mRNA. Data are presented as mean relative luminescence ± SD; *n* = 3.

Helper lipids, including a phospholipid, cholesterol, and PEGylated lipid, have been widely used in ionizable lipid mRNA formulations to improve nanoparticle stability and biodistribution and aid in endosomal escape. However, their integration within polymeric delivery systems is less common. Cationic polyesters are predominantly complexed with the mRNA, forming a polyplex or coformulated with PEG-lipids or poloxamers.^29,45,46^ Given the ability of APEs to form stable lipid–polymer hybrid nanoparticles, we set out to establish the role of helper lipids (phospholipid and cholesterol) in facilitating mRNA delivery by APE-LNPs. One of the top-performing polymers, AA2-NL-3, was formulated into APE-LNPs with Cy5-labeled FLuc mRNA and C14-PEG2000 in the presence or absence of DOPE and/or cholesterol (Table S4). In the absence of both helper lipids, we observed a 97% reduction in cellular uptake of APE-LNPs and a 120-fold reduction in FLuc mRNA expression in HeLa cells (Figures 4A–D and S11). Lipid nanoparticles formulated with a structurally different polymer (AA4-NL-3) displayed nearly identical behavior, confirming the critical role of these excipients for APE-mediated mRNA delivery (Figure S12A). Zooming in on the role of individual components, the removal of cholesterol did not significantly alter cellular uptake of AA2-NL-3-LNPs and only resulted in a 2-fold reduction in FLuc expression, suggesting that cholesterol could aid in facilitating the endosomal escape but may not be necessary for APE-LNP-mediated mRNA delivery. We found that transfection efficacy was mostly dependent on the phospholipid (DOPE), since its removal impaired the cellular uptake of the AA2-LNPs by 70% and reduced FLuc expression by 60-fold as compared to APE-LNPs containing all helper lipids. Formulations lacking DOPE displayed similar size and EE compared to four-component APE-LNPs, but their ζ-potential decreased from approximately −5 to −15 mV, which could contribute to the reduced cellular uptake across negatively charged cell membranes (Figure S10). Noting the importance of the phospholipid, we further investigated the impact of phospholipid composition on APE-LNP-mediated mRNA delivery. Phospholipid head groups and saturation of the alkyl tails were shown to have a strong impact on the bilayer-forming properties and endosomal escape of the LNP.^47^ We, therefore, compared APE-LNPs containing 1,2-dioleoyl-*sn*-glycero-3-phosphocholine (DOPC), 1,2-distearoyl-*sn*-glycero-3-phosphocholine (DSPC), or DOPE (Figure 4E,F). DOPE and DSPC have been predominantly used to formulate LNPs with ionizable lipids.^48^ DSPC enhances bilayer membrane stability relating to its high phase transition temperature, whereas DOPE has a low phase transition temperature and was reported to introduce membrane curvature and increase tension, thereby promoting inverted hexagonal phase transition.^48−50^ In comparison to DOPE, the incorporation of DSPC drastically reduced the mRNA delivery efficacy of APE-LNPs by 94% (16-fold), followed by a 30% decrease upon substitution with DOPC. These data suggest that APEs prefer interaction with unsaturated phospholipids displaying low transition temperatures (Tm (°C): DOPE −16, DOPC −17, and DSPC +55)^51^ that can promote the formation of fluid bilayers. These features aid in driving inverted hexagonal phase transition and, in turn, facilitate improved endosomal membrane fusion, allowing for more efficient mRNA delivery of APE-LNPs incorporating DOPE or DOPC as compared to DSPC.

### Polymer Side Chain Length Influences In Vivo mRNA Delivery Efficacy of APE-LNPs in Different Tissues

**Figure 5:**
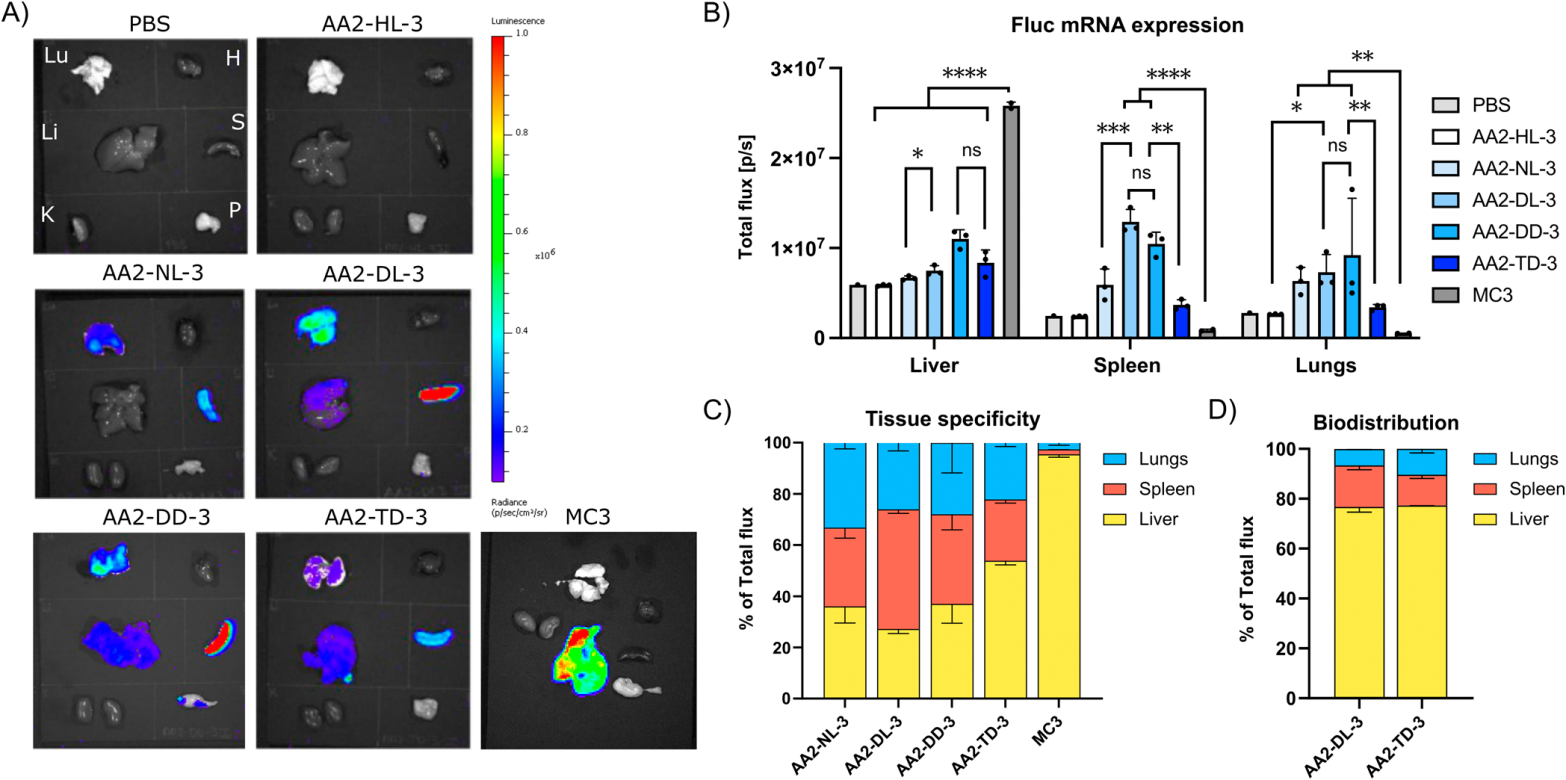
*In vivo* evaluation of APEs. C57BL/6 mice were injected via tail vein with 0.5 mg/kg of APE-LNPs containing mRNA encoding Firefly luciferase (FLuc) mRNA and imaged by IVIS after 6h. (A) Representative images of FLuc mRNA expression within the tissues; Lu: lungs, H: heart, Li: liver, S: spleen, K: kidney, P: pancreas. (B) Quantification of FLuc mRNA expression in selected tissues. Data are presented as mean ± SD; *n* = 3. Ns, *p* > 0.05, *p < 0.05, **p < 0.001, ****p < 0.0001. (C) Quantification of FLuc mRNA expression in selected organs shown as % of Total flux. Data are presented as mean ± SD; *n* = 3. (D) Quantification of DiR fluorescence expression in selected organs shown as % of Total flux. Data are presented as mean ± SD; *n* = 2.

AA2 polymers were selected for *in vivo* studies to explore the effect of polymer side chain length on the mRNA delivery and distribution of the APE-LNPs. C57BL/6 mice were injected intravenously via the tail vein with APE-LNPs containing FLuc mRNA at a 0.5 mg kg^–1^ dose. We observed that the polymer side chain length influenced the tissue selectivity and efficacy of mRNA delivery (Figure 5A). AA2-HL-3 LNPs were ineffective, corroborating our *in vitro* findings showing that a side chain longer than one carbon is needed to allow mRNA delivery.^25^ APE-LNPs with side chain lengths ranging from C4 to C9 could efficiently deliver mRNA to the lungs and spleen and target the liver, with differences in organ selectivity depending on the side chain length (Figure 5B,C). AA2-NL-3 and AA2-DL-3, with C4 and C5 side chains, respectively, predominantly delivered mRNA to the spleen and lungs. In contrast, AA2-DD-3 and AA2-TD-3, with C7 and C9 side chains, showed enhanced delivery to the liver, accompanied by a decrease in delivery to other organs (Figure 5B). Overall, mRNA delivery to the liver of AA2-DD-3 was significantly less efficient (2.4-fold) compared to liver-targeting MC3 LNPs and could be further reduced by 3.5-fold by fine-tuning the side chain composition, as demonstrated with AA2-DL-3 (Figure 5B). This emphasizes the potential to develop APEs as an mRNA delivery platform to nonliver tissues. Liver targeting could potentially be further reduced by excluding cholesterol from APE-LNP formulations due to their low reliance on this excipient for mRNA delivery, as shown *in vitro* (Figure 4B). This strategy was recently employed by Su et al., to decrease liver accumulation for LNPs containing permanently charged lipids.^52^ Based on our findings, we also investigated if the 4C side chain can improve mRNA delivery of the previously reported lung-targeting AA3-DD-3 LNP with a 7C side chain and found that reducing side chain length resulted in a 2.4-fold increase in mRNA delivery to the lungs (Figure S13). This reinforces the importance of optimizing the side chain composition to improve the *in vivo* performance of APE-LNPs.^25^ Optimizing the hydrophobicity of polyesters by Yu et al. led to enhanced performance of mRNA polyplexes in the lungs, suggesting the contribution of polymer hydrophobic domains in interactions with serum or cell components, although the mechanism remains unclear.^53^ Notably, the effect of changes in hydrophobicity on the ability of LNPs to target specific organs may differ between biomaterials. Liu et al. reported that multitailed ionizable phospholipids (iPhos) with short alkyl tails predominantly targeted the liver, while long tails preferentially targeted the spleen.^54^

To gain further insights into the differences in mRNA delivery to different organs, we studied the biodistribution of APE-LNPs labeled with the fluorescent dye DiR. We compared polymers with a 4C side chain (AA2-DL-3), which exerted selectivity to the lungs and spleen, and the long (C9) side chain (AA2-TD-3), which showed more mRNA delivery to the liver (Figure 5B). The measurement of DiR fluorescence showed that, in both cases, the majority of APE-LNPs accumulated in the liver (±75%) with lower distribution to the spleen (10–15%) and lungs (5–10%) (Figures 5D and S14A,B). Our biodistribution data indicate that there is no direct correlation between APE-LNP uptake by certain tissues and functional mRNA delivery. However, these results corroborate other findings with lipid and polymeric nanoparticles, where organs with the highest accumulation did not exhibit the highest mRNA expression.^7,25,53^ We speculate that organ selectivity may relate to alterations in the formation of the protein corona resulting from the alkyl side chain’s impact on the chemical composition and physicochemical properties of APE-LNPs, but detailed mechanistic studies are required to confirm this hypothesis.^55,56^ Further studies are needed to determine the likely complex mechanism behind this *in vivo* behavior of both lipid and polymer delivery systems.

The apparent acid dissociation constant (p*K*_a_) of LNPs containing ionizable lipids was shown to affect mRNA delivery to different organs.^11^ We therefore studied the impact of the APE alkyl side chain composition on the p*K*_a_ of APE-LNPs using 2-(*p*-toluidino)naphthalene-6-sulfonic acid (TNS) assay.^57^ The apparent p*K*_a_ of APE-LNPs ranged from 3 to 6 and was not significantly affected by the length of the polymer side chain (Tables 1, S2, and Figure S9A,B), but rather mainly driven by the composition of the tertiary amino-alcohol. AA2-LNPs evaluated *in vivo* exhibited comparable p*K*_a_ between 4 and 5 (Figure S15D), which could potentially explain their inefficient mRNA delivery to the liver despite accumulation in liver tissue, as the optimal p*K*_a_ reported for liver-targeting LNPs is between 6.2 and 6.5.^41^ However, the link between p*K*_a_ and mRNA delivery to nonliver tissues has not been well established. As shown by Dilliard et al., LNP formulations including charged lipids with p*K*_a_ between 2 and 6 tend to target the spleen, while with p*K*_a_ above 7 tend to deliver to the lungs, which does not correspond with our findings.^11^ As indicated above, the polymer and lipid nanoparticle composition may also be relevant for interaction with key serum components influencing the formation of the protein corona on the nanoparticle surface, which needs to be further investigated.^58−60^

## Conclusion

In this study, we show the impact of APE alkyl side chain composition on the design of APE-LNPs for mRNA delivery to nonliver tissues and demonstrate the potential of low molecular weight APEs to be developed as an alternative to ionizable lipids in LNP formulation. Our findings reveal that the optimal alkyl side chain length is critical for the assembly of stable mRNA nanoparticles and their ability to efficiently deliver mRNA both *in vitro* and *in vivo*. Tissue selectivity of mRNA APE-LNPs is also impacted by the polymer alkyl side-chain length, where polymers with short carbon side chains (4–5 carbons) target the spleen and lungs more effectively than polymers with longer side chains (7–9 carbons), which show increased mRNA delivery to the liver. We also unraveled the relevance of helper lipids in APE-LNP formulation, in particular the critical role of the unsaturated phospholipids in cellular uptake and mRNA delivery efficacy, as well as the low dependency of APE-LNPs on cholesterol, which may be further leveraged to reduce liver uptake. Altogether, our findings provide valuable insights into the design of polymers for use in the context of LNPs, which can help identify alternatives to ionizable lipids and expand the therapeutic utility of mRNA LNPs beyond the liver.

## Materials and Methods

### Materials

δ-Hexanolactone (HL) was purchased from Fisher Scientific Ireland. δ-Nonalactone (NL), 5-decanolide (DL), jasmolactone (JL), δ-dodecalactone (DD), δ-tetradecalactone (TD), *N*,*N*,*N*′,*N*′-tetrakis(2-hydroxyethyl)ethylenediamine amines used for the synthesis of the amino-alcohols 3,3′-diamino-*N*-methyldipropylamine, tris[2-(methylamino)ethyl]amine, *N,N*-dimethylethylenediamine, *N,N’*-dimethylethylenediamine, 1,4,7-triazacyclononaneand, and the catalyst 1,5,7-triazabicyclo[4.4.0]dec-5-ene (TBD) were purchased from Sigma-Aldrich Ireland. Custom amino-alcohols, 3,3′,3′’,3′’’-(((methylazanediyl)bis(propane-3,1-diyl))bis(azanetriyl))tetrakis(propan-1-ol), 3,3′,3′’-((nitrilotris(ethane-2,1-diyl))tris(methylazanediyl))tris(propan-1-ol), 3,3′-((2-(dimethylamino)ethyl)azanediyl)bis(propan-1-ol), 3,3′-(ethane-1,2-diylbis(methylazanediyl))bis(propan-1-ol), and 3,3′,3′’-(1,4,7-triazonane-1,4,7-triyl)tris(propan-1-ol) were synthesized according to the procedure described below. All the solvents were purchased from Sigma-Aldrich Ireland at ACS grade. All chemical reagents were used as received with no further purification. The benchmark ionizable lipids 1-[2-[bis(2-hydroxydodecyl)amino]ethyl-[2-[4-[2-[bis(2-hydroxydodecyl)amino]ethyl]piperazin-1-yl]ethyl]amino]dodecan-2-ol (C12–200), and [(2R)-2,3-di(tetradecanoyloxy)propyl] 2-(trimethylazaniumyl)ethyl phosphate (MC3) were purchased from BroadPharma (San Diego, CA) and CordenPharma (Basel, Switzerland), respectively. The lipids 1,2-dioleoyl-*sn*-glycero-3-phosphoethanolamine (DOPE), 1,2-di(9Z-octadecenoyl)-*sn*-glycero-3-phosphocholine (DOPC), 1,2-dioctadecanoyl-*sn*-glycero-3-phosphocholine (DSPC), 1,2-Dimyristoyl-rac-glycero-3-methoxypolyethylene glycol-2000 (DMG-PEG_2000_) 1,2-dimyristoyl-*sn*-glycero-3- phosphoethanolamine-*N*-[methoxy-(polyethylene glycol)-2000] (ammonium salt) (C14-PEG_2000_) and the sterol cholesterol were purchased from Avanti. The TNS reagent 2-(*p*-toluidino)naphthalene-6-sulfonic acid was purchased from Sigma-Aldrich Ireland. The fluorescent dye 1,1′-dioctadecyl-3,3,3′,3′-tetramethylindotricarbocyanine Iodide (DiR) was purchased from Thermo Fisher Scientific Ireland. Lipofectamine MessengerMax transfection reagent was purchased from Fisher Scientific Ireland. Cy5-labeled luciferase mRNA was purchased from TriLink BioTechnologies.

### Instrumentation and Characterization

Molecular weight and polydispersity (Đ) of the polymers were determined by gel permeation chromatography (GPC) using a Tosoh EcoSEC HLC-8320GPC with a refractive index (RI) detector, conducted in tetrahydrofuran (THF) mobile phase, calibrated with linear polystyrene standards on a TKSgel G3000 + 4000HHR column operating at 1.0 mL min^–1^. Samples were filtered through 0.45 μm PTFE filters (Fisher Scientific Ireland) before injection, at an approximately 2 mg mL^–1^ polymer concentration.

^1^H NMR spectra were recorded on a Bruker 400 MHz NMR spectrometer in deuterated chloroform (CDCl_3_, Sigma-Aldrich Ireland), using as internal reference the residual proton resonance of the solvent peak at 7.26 ppm. ^13^C NMR spectra were recorded on a Bruker 400 MHz NMR spectrometer in deuterated chloroform (CDCl_3_), using the residual proton resonance of the solvent peak at 77.160 ppm as an internal reference. Chemical shifts (δ) are reported in parts per million (ppm). Splitting patterns are reported as follows: singlet (s), doublet (d), triplet (t), quadruplet (q), quintuplet (quint), and multiplet (m). All NMR spectra were processed using MestReNova NMR software, version 12.0.0–20080 (Mestrelab Research S.L.).

Nominal mass spectra were recorded on a Waters Quattro Micro triple quadrupole instrument in electrospray ionization (ESI) mode using 50% acetonitrile–water containing 0.1% formic acid as the eluent; samples were prepared at a concentration of approximately 1 mg mL^–1^ in water.

### Synthesis of the Custom Amino-Alcohols

The synthesis of the custom amino alcohols was performed as previously reported by Kowalski et al.^25^

As an example, 3,3′-diamino-*N*-methyldipropylamine (2.22 mL, 13.8 mmol, 1 equiv) was dissolved in dry MeOH (10 mL). Methyl acrylate (6.2 mL, 68.9 mmol, 5 equiv) was added to the solution dropwise. The reaction mixture was stirred at room temperature (RT) under an inert atmosphere for 2 days. The completion of the reaction was analyzed by ^1^H NMR. The solvent was removed under reduced pressure and purified by column chromatography in silica gel (pore size of 60 Å, 230–400 mesh particle size, and 40–63 μm particle size) using DCM/MeOH/NH_4_OH (87.5:11:1.5) as the mobile phase. The reaction mixture was dissolved in the minimum amount of mobile phase and added to the column when the silica was compacted. The collected fractions were checked by thin-layer chromatography (TLC), and the fractions containing the purified amino-alcohol were mixed. The solvents were removed under reduced pressure, and the purified product was taken to the next step. A round-bottomed flask containing the ester was purged with N_2_ and dissolved in anhydrous THF (12 mL). The reaction flask was cooled to 0 °C, and 1 M LiAlH_4_ in THF (Sigma-Aldrich Ireland) (47.7 mL, 1256.1 mmol, 4.2 equiv) was added dropwise. The reaction mixture was equilibrated to RT overnight. Na_2_SO_4_·10 H_2_O and 2-methyltetrahydrofuran (10 mL) were added to quench the reaction and stirred for 30 min until all gray solids turned into a white suspension. Next, the mixture was filtered using a Büchner funnel with filter paper (grade 3) covered with anhydrous Na_2_SO_4_ to ensure the removal of solids and water. If the filtrate appeared cloudy, additional drying with Na_2_SO_4_ and filtration with a PTFE 0.45 μm filter were performed. The filtrate was evaporated under reduced pressure to yield the amino-alcohol AA2 (2.9 g, 76%) as a pale-yellow syrup. AA3, AA4, AA6, and AA7 were similarly synthesized, and the resulting yields for all the AAs are presented in Table S5. All of the synthesized amino-alcohols were characterized by NMR and ESI (characterization of amino-alcohols, Supporting Information).

### Synthesis of Amino-Polyesters (APEs)

APEs were synthesized via ring-opening polymerization (ROP) of the different lactones (HL, NL, DL, JL, DD, and TD) in the presence of an amino-alcohol (AA1, AA2, AA3, AA4, AA6, AA7) as an initiator and TBD as a catalyst in bulk at room temperature. The monomer-to-initiator hydroxyl group ratio was set equal to 3 in order to obtain APEs with 3 units of lactones for each arm. The hydroxyl group of the initiator-to-catalyst molar ratio was set to be equal to 10, 6.3, or 4.4, depending on the lactone used. As an example, for AA1-HL-3, 1.159 g of HL, 0.2 g of AA1, and 24 mg of Na_2_SO_4_ anhydrous were poured into a 10 mL vial and left to stir for 15 min. Then, the mixture was poured into another vial with 47 mg of TBD and 24 mg of Na_2_SO_4_ anhydrous and was left to react under vigorous stirring for 24 h at room temperature. The polymerization was stopped by adding an excess of benzoic acid in dichloromethane or diethyl ether (1 mmol mL^–1^). The final mixture was further diluted in dichloromethane and washed three times with a saturated solution of NaCl. The organic phase was recovered, dried with Na_2_SO_4_ anhydrous, filtered with a PTFE 0.45 μm filter, and the solvent was removed under reduced pressure to obtain the purified APE as a viscous syrup. The final APEs were characterized via GPC and ^1^H NMR (CDCl_3_, Bruker, 400 MHz) before and after purification.

### Differential Scanning Calorimetry (DSC)

Differential scanning calorimetry (DSC) was employed to determine the glass transition temperature (Tg) of the APEs. A DSC Q1000 (TA Instruments) was used, and the thermograms were analyzed using TA Universal Analysis software. Weighted samples (3–10 mg) were measured in hermetically sealed pans; after sealing the pans, a hole was made to allow solvent traces to evaporate. The samples were subjected to 3 heating and cooling cycles with a ramp from 120 to −80 °C at 10 °C min^–1^. The first heating cycle was used to evaporate any traces of the solvent. Tg was determined as the midpoint between the onset and end of the change in specific heat observed as a shift in the baseline in the thermogram of a dynamic scan.

### mRNA Synthesis and Characterization

DNA plasmids containing a T7 promoter upstream of the sequence encoding for luciferase (FLuc) were used as templates for mRNA synthesis. The DNA plasmids were linearized using the restriction enzyme XbaI (New England Biolabs, Ipswich, MA) and transcribed using the HiScribe T7 RNA Synthesis Kit (New England Biolabs). To synthesize nucleoside-modified mRNA, uridine triphosphate was replaced with either 5-methoxyuridine (5moU) triphosphate or N1-methylpseudouridine (m1ψ) triphosphate (TriLink, San Diego, CA) in the transcription reaction. The mRNA was post-transcriptionally capped using the Vaccinia Capping System (New England Biolabs) and mRNA Cap2′-*O*-Methyltransferase (New England Biolabs) resulting in a Cap1 structure. A poly(A) tail of approximately 120 nucleotides was added by using *E. coli* Poly(A) Polymerase (New England Biolabs). All mRNAs were purified using the Monarch RNA Cleanup Kit (New England Biolabs). RNA concentration was determined using a NanoDrop One (Thermo Scientific). The final purified mRNAs contained a 5′ cap (Cap1), 5′ and 3′ UTRs derived from the human hemoglobin subunit beta (HBB) gene, a coding region as listed below, and a poly(A)tail. mRNA integrity and purity were characterized by agarose gel electrophoresis under denaturing conditions (E-Gel 2% EX gels, ThermoFisher). The gels were imaged using an iBright Imaging System (ThermoFisher).

FLuc:

ATGGAAGATGCCAAAAACATTAAGAAGGGCCCAGCGCCATTCTACCCACTCGAAGACGGGACCGCCGGCGAGCAGCTGCACAAAGCCATGAAGCGCTACGCCCTGGTGCCCGGCACCATCGCCTTTACCGACGCACATATCGAGGTGGACATTACCTACGCCGAGTACTTCGAGATGAGCGTTCGGCTGGCAGAAGCTATGAAGCGCTATGGGCTGAATACAAACCATCGGATCGTGGTGTGCAGCGAGAATAGCTTGCAGTTCTTCATGCCCGTGTTGGGTGCCCTGTTCATCGGTGTGGCTGTGGCCCCAGCTAACGACATCTACAACGAGCGCGAGCTGCTGAACAGCATGGGCATCAGCCAGCCCACCGTCGTATTCGTGAGCAAGAAAGGGCTGCAAAAGATCCTCAACGTGCAAAAGAAGCTACCGATCATACAAAAGATCATCATCATGGATAGCAAGACCGACTACCAGGGCTTCCAAAGCATGTACACCTTCGTGACTTCCCATTTGCCACCCGGCTTCAACGAGTACGACTTCGTGCCCGAGAGCTTCGACCGGGACAAAACCATCGCCCTGATCATGAACAGTAGTGGCAGTACCGGATTGCCCAAGGGCGTAGCCCTACCGCACCGCACCGCTTGTGTCCGATTCAGTCATGCCCGCGACCCCATCTTCGGCAACCAGATCATCCCCGACACCGCTATCCTCAGCGTGGTGCCATTTCACCACGGCTTCGGCATGTTCACCACGCTGGGCTACTTGATCTGCGGCTTTCGGGTCGTGCTCATGTACCGCTTCGAGGAGGAGCTATTCTTGCGCAGCTTGCAAGACTATAAGATTCAATCTGCCCTGCTGGTGCCCACACTATTTAGCTTCTTCGCTAAGAGCACTCTCATCGACAAGTACGACCTAAGCAACTTGCACGAGATCGCCAGCGGCGGGGCGCCGCTCAGCAAGGAGGTAGGTGAGGCCGTGGCCAAACGCTTCCACCTACCAGGCATCCGCCAGGGCTACGGCCTGACAGAAACAACCAGCGCCATTCTGATCACCCCCGAAGGGGACGACAAGCCTGGCGCAGTAGGCAAGGTGGTGCCCTTCTTCGAGGCTAAGGTGGTGGACTTGGACACCGGTAAGACACTGGGTGTGAACCAGCGCGGCGAGCTGTGCGTCCGTGGCCCCATGATCATGAGCGGCTACGTTAACAACCCCGAGGCTACAAACGCTCTCATCGACAAGGACGGCTGGCTGCACAGCGGCGACATCGCCTACTGGGACGAGGACGAGCACTTCTTCATCGTGGACCGGCTGAAGTCCCTGATCAAATACAAGGGCTACCAGGTAGCCCCAGCCGAACTGGAGAGCATCCTGCTGCAACACCCCAACATCTTCGACGCCGGGGTCGCCGGCCTGCCCGACGACGATGCCGGCGAGCTGCCCGCCGCAGTCGTCGTGCTGGAACACGGTAAAACCATGACCGAGAAGGAGATCGTGGACTATGTGGCCAGCCAGGTTACAACCGCCAAGAAGCTGCGCGGTGGTGTTGTGTTCGTGGACGAGGTGCCTAAAGGACTGACCGGCAAGTTGGACGCCCGCAAGATCCGCGAGATTCTCATTAAGGCCAAGAAGGGCGGCAAGATCGCCGTGTAA

### Formulation and Characterization of the Nanoparticles

The APE lipid nanoparticles (APE-LNPs) were formulated by microfluidics, mixing ethanol and aqueous phases at a 1:3 volumetric ratio using syringe pumps (Pump 33 DDS, Harvard Apparatus), as previously described.^43^ The ethanol phase was prepared by solubilizing a mixture of ionizable APE, DOPE, cholesterol, and C14-PEG_2000_ at a molar ratio of 50:25:23.5:1.5 and A 9:1 nitrogen-to-phosphate (N/P) ratio. The aqueous phase contained mRNA suspended in 10 mM citrate buffer at pH 3.2. The APE-LNPs formulated with DOPC and DSPC were prepared by substituting DOPE. The APE-LNPs labeled with DiR were formulated as described above, with an ethanol phase composed of APE, DOPE, cholesterol, DiR, and C14-PEG_2000_ at a molar ratio of 50:25:22.5:1:1.5, and the same aqueous phase composition. The concentration of mRNA encapsulated into APE nanoparticles was determined by the Quant-iT RiboGreen assay (ThermoFisher), according to the manufacturer’s protocol. The efficacy of mRNA encapsulation into APE-LNPs was calculated by comparing measurements in the absence and presence of 1% (v/v) Triton X-100. Nanoparticle size, polydispersity (PDI), and ζ-potential (ZP) were analyzed by dynamic light scattering (DLS) using a Zetasizer Nano ZS (Malvern Instruments, Worcestershire, UK). APE-LNP hydrodynamic diameters are reported as the average Z-average of at least two independent measurements. The stability of the APE-LNPs was evaluated by measuring the size of the particles stored at 4 °C at days 0, 7, 14, and 28 using DLS, as described above.

C12–200 lipid nanoparticles were formulated by microfluidics, mixing ethanol and aqueous phases at a 1:3 volumetric ratio using syringe pumps, as previously described. The ethanol phase was prepared by solubilizing a mixture of C12–200, DOPE, cholesterol, and C14-PEG_2000_ at a molar ratio of 35:16:46.5:2.5 and a 10:1 Nitrogen-to-Phosphate (N/P) ratio. The aqueous phase contained mRNA suspended in 10 mM citrate buffer at pH 3.2. The concentration of encapsulated mRNA, as well as the size, PDI, and ZP, were analyzed as described above.

MC3 lipid nanoparticles were formulated by microfluidics, mixing ethanol and the aqueous phase at a 1:3 volumetric ratio using syringe pumps, as previously described. The ethanol phase was prepared by solubilizing a mixture of MC3, DSPC, cholesterol, and DMG-PEG_2000_ at a molar ratio of 50:10:38.5:1.5 and a 14:1 nitrogen-to-phosphate (N/P) ratio. The aqueous phase contained mRNA suspended in 10 mM citrate buffer at pH 3.2. The concentration of encapsulated mRNA, as well as the size, PDI, and ZP, were analyzed as described above.

### APE-LNP p*K*_a_ and Surface Ionization Measurements

Nanoparticle surface p*K*_a_ was determined using the 2-(*p*-toluidino)naphthalene-6-sulfonic acid (TNS) assay. Briefly, 20 mM citrate, PBS, and ammonium acetate buffers were prepared and titrated to pH values ranging from 1 to 12 in increments of 0.5. The citrate buffer was used from pH 1 to 6, PBS from pH 6.5 to 7.5, and ammonium acetate from pH 8 to 12. The pH of the buffers was adjusted using 1 M sodium hydroxide and 1 M hydrochloric acid. A 300 mM stock of 6-(*p*-toluidino)-2-naphthalenesulfonic acid sodium salt (TNS reagent) was prepared in DMSO, and APE-LNPs were diluted to 50 μM total lipids in PBS. 88 μL of buffer, 10 μL of APE-LNPs, and 2 μL of TNS reagent were added to a black 96-well plate in duplicates. The plate was incubated in the dark, shaking at 100 rpm for 5 min at room temperature. TNS fluorescence intensity was measured at λ_ex_ = 322 nm and λ_em_ = 431 nm using a Tecan SPARK plate reader (Tecan, Reading, UK). The APE-LNP surface p*K*_a_ was determined to be the pH at which 50% of the amine proportion occurred. All measurements were performed using a manual gain setting of 45.

### Cryogenic Transmission Electron Microscopy (Cryo-TEM)

For Cryogenic Transmission Electron Microscopy (cryo-TEM) samples (3 μL) were applied to glow-discharged Lacey carbon film on 300 Cooper mesh (Ted Pella 01896-F) and vitrified in liquid ethane using an FEI Vitrobot Mark IV (Thermo Fisher Scientific) at 4 °C with 95% humidity, a 4 s blot time, and 0 blot force. Grids were imaged with a Glacios electron microscope (Thermo Fisher Scientific) operating at 200 kV and equipped with a Falcon 3EC Direct Electron Detector at the Centre of New Technologies, University of Warsaw. Images were recorded in linear mode with a physical pixel size of 1.586 Å (nominal magnification of 92,000x) and a total dose of 27.19 e/Å^2^.

### Cell Culture

HeLa cells (provided by Dr. Krajewska’s lab) were cultured in Dulbecco’s Modified Eagle’s Medium (DMEM) (containing 4500 mg L^–1^ glucose, l-glutamine, sodium pyruvate, and sodium bicarbonate) (Cat No. D6429, Merck) supplemented with 10% heat-inactivated fetal bovine serum (hiFBS, Merck) and 1% penicillin/streptomycin (Merck). Cells were passaged every 3–4 days.

The human monocyte cell line THP-1 was donated by the Cancer Research @UCC research center, University College Cork. Cells were cultured in Roswell Park Memorial Institute (RPMI) medium 1640 (containing 4500 mg L^–1^d-glucose, 2383 mg L^–1^ HEPES buffer, l-glutamine, 110 mg L^–1^ sodium pyruvate, and 1500 mg L^–1^ sodium bicarbonate (Cat No. A1049101, Fisher Scientific), supplemented with 10% hiFBS and 1% penicillin/streptomycin. Cells were passaged every 2–3 days.

The human umbilical vein endothelial cells (HUVECs) were purchased from Lonza Bioscience (Basel, Switzerland). The cells were cultured in EBM-2 basal medium supplemented with the EGM-2 MV SingleQuots Kit Supplements & Growth Factors (Lonza Bioscience). HUVECs from passages 4 to 6 were used for experiments.

All cell lines were incubated in a humidified atmosphere of 5% CO_2_ at 37 °C and routinely tested for the absence of mycoplasma using the Mycoplasma Detection Kit (Jena Bioscience, Thüringen, Germany, Cat No. PP-401).

### In Vitro Transfections

For the APE library screen, HeLa and HUVEC cells were seeded in white 96-well plates (Costar) at 10,000 cells per well 1 day before the experiment. THP-1 cells were seeded in white 96-well plates at 20,000 cells per well and stimulated with phorbol ester, phorbol-12-myristate-13-acetate (PMA), at a concentration of 10 ng mL^–1^ 2 days before the experiment to differentiate the cells into an M0 phenotype. One day before the experiment, the media was changed to remove the PMA. APE-LNPs, C12–200, and MC3 nanoparticles containing 50 ng of 5moU-modified FLuc mRNA were added to each well, and the cells were incubated for 24 h. Cell viability and FLuc expression were analyzed with CellTiter-Fluor Cell Viability and One-Glo Luciferase assays (Promega, distributed by MyBio), according to the manufacturer’s protocol. The fluorescence and luminescence signals were quantified using a Tecan SPARK plate reader (Tecan, Reading, UK).

### Cellular Uptake Analysis

For the flow cytometry analysis, HeLa cells were seeded at 30,000 cells/cm^2^ into 24-well plates 1 day before the experiment and incubated for 24 h with the APE-LNPs containing 250 ng of Cy5-labeled 5moU-modified FLuc mRNA (TriLink BioTechnologies). Cells were washed with PBS and detached from the surface using TrypLE Express Enzyme (Thermo Fisher, Ireland), after which they were immediately transferred to tubes containing 5% FBS in PBS and kept on ice. Next, samples were centrifuged for 5 min at 400*g* at 4 °C, followed by two washing steps with 5% FBS in PBS and resuspended in 0.2 mL of PBS containing 0.5% paraformaldehyde (PFA) to fix the cells. Samples were analyzed using a BD FACSCelesta flow cytometer (BD Biosciences), and analysis was performed with FlowJo v10.4 software.

For fluorescent microscopy analysis, HeLa cells were seeded onto cover glasses in a 12-well plate at 100,000 cells per well 1 day before the experiment. Cells were incubated for 24 h with the APE-LNPs containing 250 ng of Cy5-labeled mRNA. Subsequently, the cells were washed with PBS and fixed in 2% PFA for 20 min. After fixation, the cells were washed and incubated with 1 μgmL^–1^ DAPI nuclear stain (Merck) for 15 min. Cover glasses were washed, mounted onto microscope slides, and stored at 4 °C. Images were captured using a confocal laser scanning microscope (Olympus FV1000) fitted with 405 and 633 nm lasers, and a 60x dry objective lens. All images were acquired in the linear range, avoiding local saturation, at an image resolution of 1024 × 1024 pixels and with a pinhole size of 1 Airy unit. Images were analyzed and processed using ImageJ v2.14.0 software.

### Animal Studies

C57BL/6 mice were provided by Envigo RMS (UK). All animals were housed and cared for in compliance with protocols and procedures approved by the Animal Experimentation Ethics Committee of University College Cork and the Health Products Regulatory Authority (HPRA) (Project Authorization Number: AE19130/P164). For mRNA delivery studies, selected APE-LNPs containing 5moU-modified FLuc mRNA were injected intravenously into female C57BL/6 mice (18–22 g) via the tail vein (0.5 mg kg^–1^). After 6 h of the injection, d-Luciferin Potassium (PerkinElmer) at a concentration of 30 mg/mL was injected intraperitoneally, and the mice were euthanized 10 min later. The organs were extracted and imaged using an IVIS Lumina Series III In Vivo Imaging System (PerkinElmer). The luminescence signals were quantified using Living Image software v4.5.2 (PerkinElmer).

For biodistribution studies, APE-LNPs labeled with DiR dye were injected intravenously into female C57BL/6 mice (18–22 g) via the tail vein (0.5 mg kg^–1^). After 6 h of the injection, the mice were euthanized, and the organs were extracted and imaged using the IVIS Lumina Series III In Vivo Imaging System (PerkinElmer). The fluorescence signals were quantified using Living Image software v4.5.2 (PerkinElmer).

### Statistical Analysis

All data were analyzed with GraphPad Prism 9 (La Jolla, CA, USA) and are presented as mean ± SD. Statistical analysis was performed by using one-way ANOVA followed by Tukey’s or Dunnett’s multiple comparisons test to compare multiple replicate means, or an unpaired two-tailed Student’s *t*-test assuming equal variances to compare two replicate means. Differences were considered significant when *p* ≤ 0.05. Correlation analysis to assess the relationship between the hydrodynamic diameter, surface charge, or mRNA encapsulation efficacy and the lactone side chain length was determined by computing a two-tailed nonparametric Spearman correlation with a 95% confidence interval. A correlation coefficient (ρ) of ρ = 1 indicates a perfect positive correlation between the variables, ρ = −1 indicates a perfect negative correlation between the variables, and ρ = 0 indicates no correlation between the variables.

## Supporting Information Available

Polymer characterization (1H-NMR, GPC, and DSC spectra) and additional nanoparticle characterization of APE-LNP formulations (including formulation composition, size, polydispersity, surface charge, p*K*_a_, encapsulation efficiency, nanoparticle uptake, mRNA delivery efficacy, biodistribution, and stability) (PDF)
