# Supplementary material for: Tailoring Alkyl Side Chains of Ionizable Amino-Polyesters for Enhanced In Vivo mRNA Delivery
Source: ACS Appl Bio Mater. 2025 Apr 28;8(5):3958–71. doi: 10.1021/acsabm.5c00116 (PMC12093369; doi:10.1021/acsabm.5c00116)
Supplement: Supplementary file 1 — mt5c00116_si_001.pdf [file mt5c00116_si_001.pdf]

Supporting information for

Tailoring Alkyl Side Chains of Ionizable Amino-Polyesters for Enhanced In Vivo mRNA Delivery

Aida López Espinar[1#], Lianne M. Mulder[1#], Mohamed Elkhashab[1], Zahra Khan[1], Mariusz Czarnocki-Cieciura[2], Maria R. Aburto[3,4], Sonja Vucen[5], and Piotr S. Kowalski[1,3,*]

1. School of Pharmacy, University College Cork, Cork, T12 K8AF, Ireland
2. Laboratory of Protein Structure, International Institute of Molecular and Cell Biology, Warsaw, 02-109, Poland
3. APC Microbiome Ireland, University College Cork, Cork, T12 K8AF, Ireland
4. Department of Anatomy and Neuroscience, University College Cork, Cork, T12 K8AF, Ireland
5. SSPC, Research Ireland Centre for Pharmaceuticals, School of Pharmacy, University College Cork, Cork T12 K8AF, Ireland

* piotr.kowalski@ucc.ie

Figure S1. 1H-NMR characterization of A) AA1-APEs (not purified), B) AA2-APEs purified (representative proton assignment for AA2-NL-3), C) AA3-APEs (AA3-JL-3 and AA3-DD-3 not purified), D) AA4-APEs (AA4-JL-3 and AA4-DL-3 not purified), E) AA6-APEs (AA6-JL-3 not purified), F) AAF-APEs (AA7-DL-3 not purified).

A)

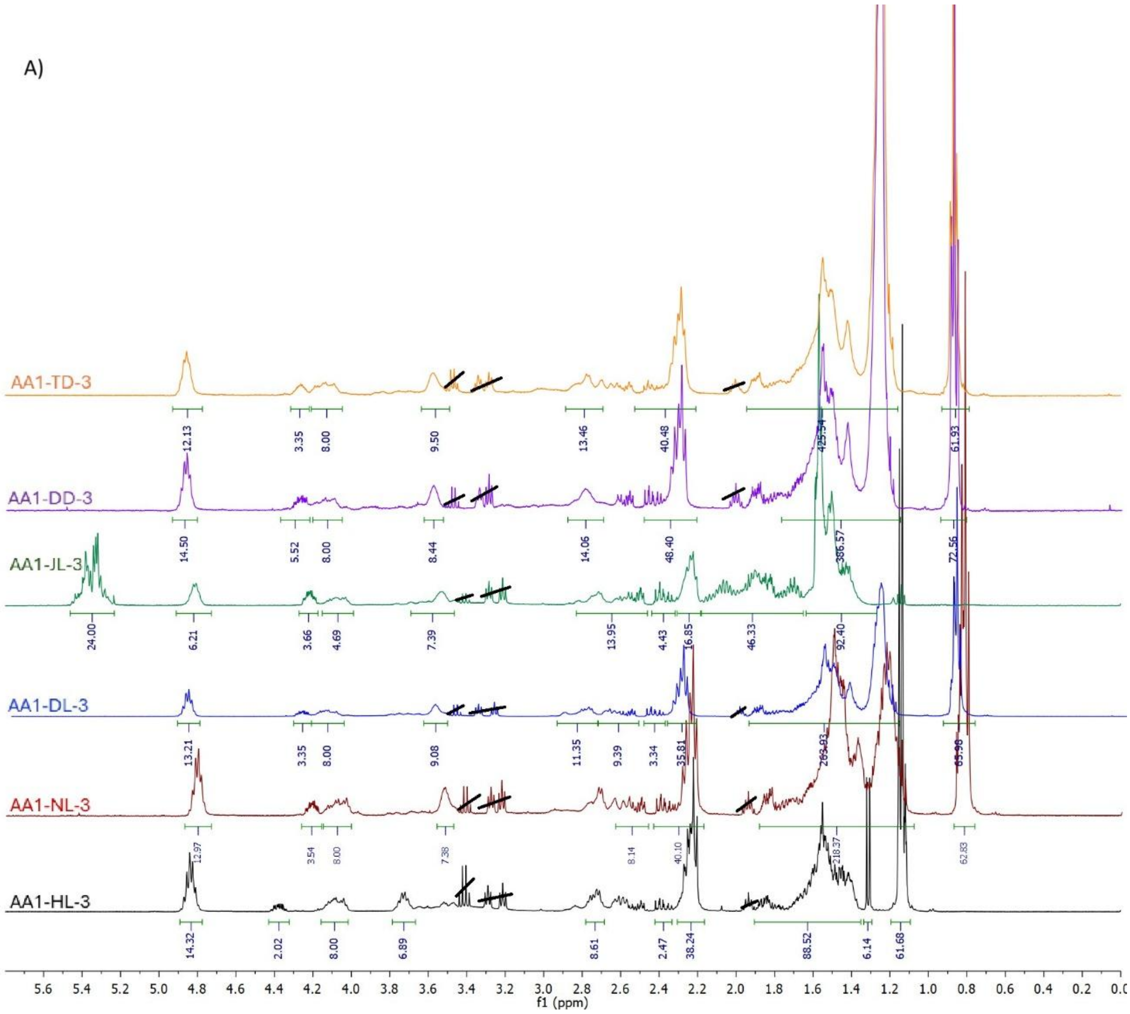

B)

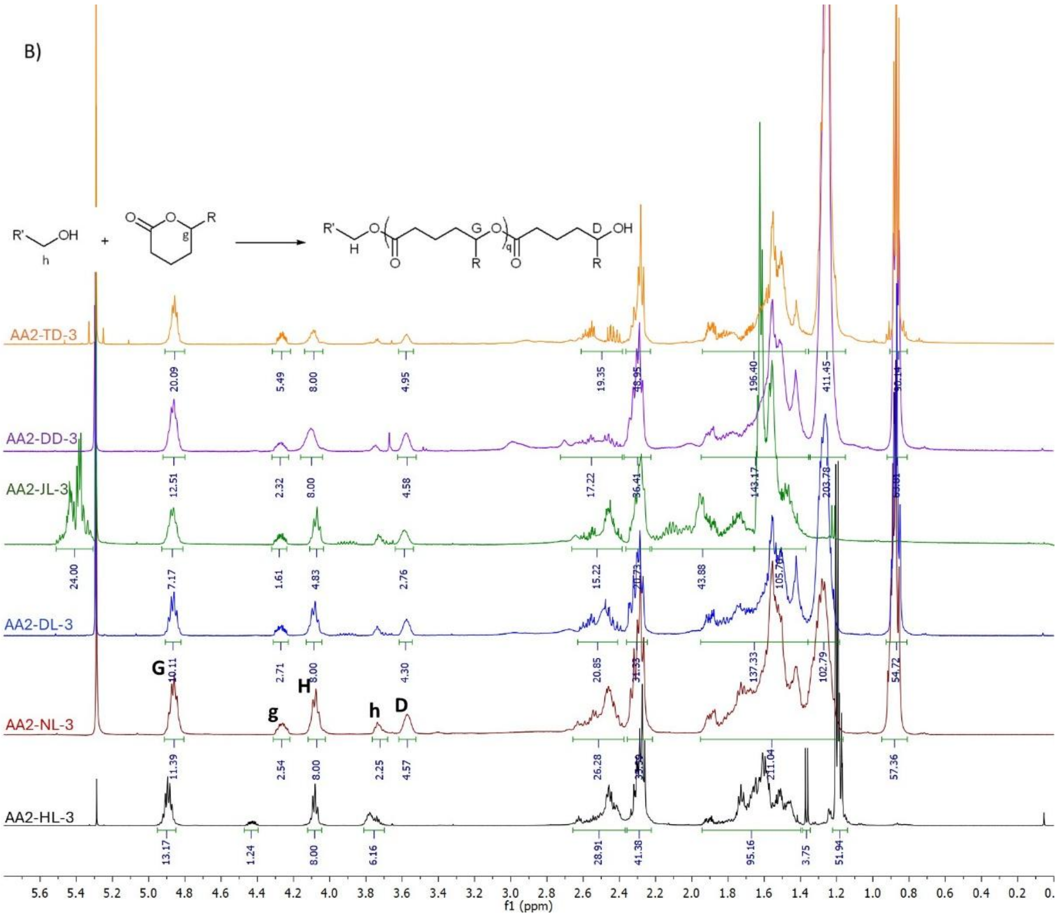
R'
OH
h
+
g
R
R'
H
G
R
q
D
OH
R
AA2-TD-3
AA2-DD-3
AA2-JL-3
AA2-DL-3
AA2-NL-3
AA2-HL-3
G
g
H
h
D
f1 (ppm)

C)

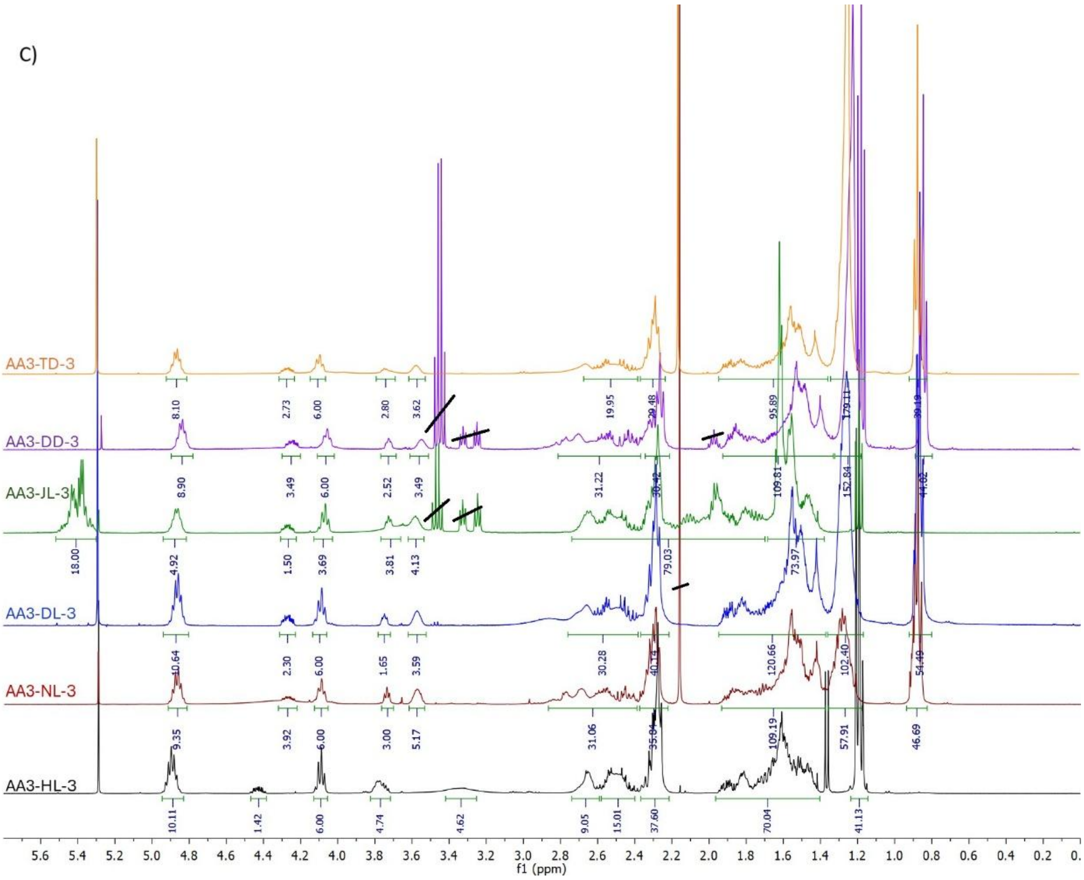
AA3-TD-3
AA3-DD-3
AA3-JL-3
AA3-DL-3
AA3-NL-3
AA3-HL-3
f1 (ppm)

D)

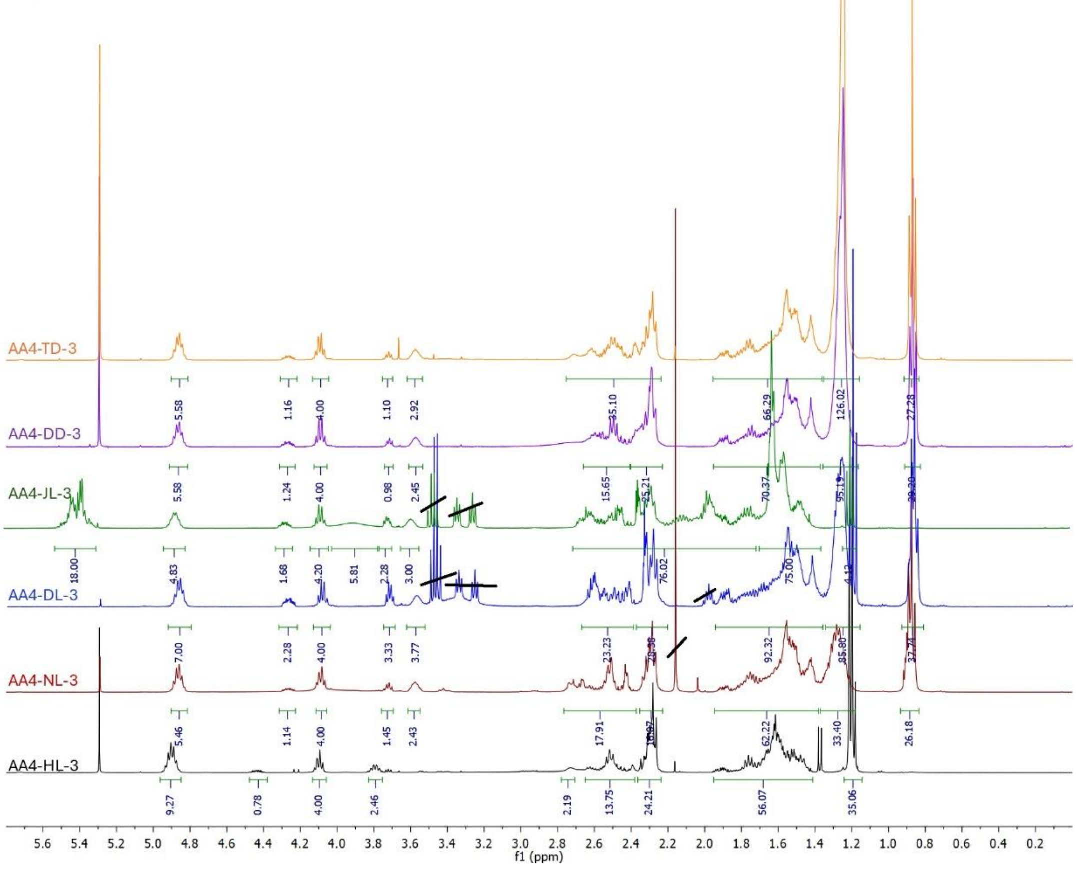
AA4-TD-3
AA4-DD-3
AA4-JL-3
AA4-DL-3
AA4-NL-3
AA4-HL-3
f1 (ppm)

E)

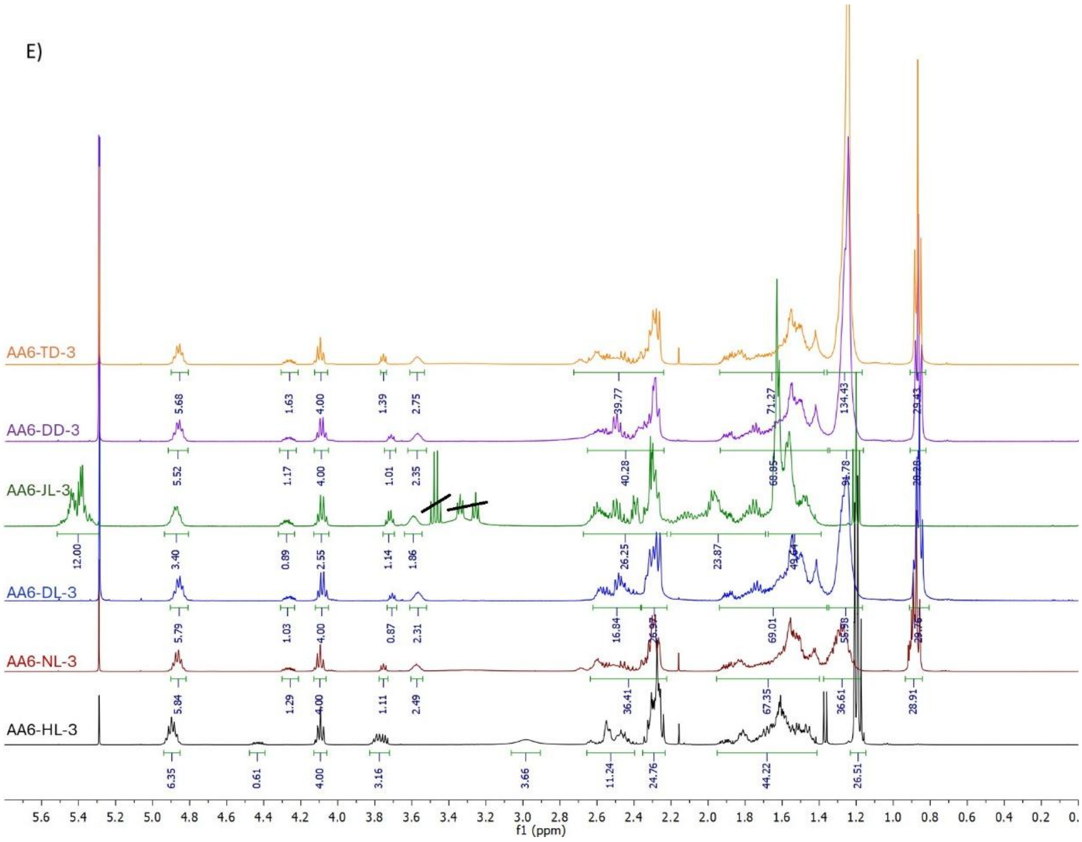
AA6-TD-3
AA6-DD-3
AA6-JL-3
AA6-DL-3
AA6-NL-3
AA6-HL-3
f1 (ppm)

F)

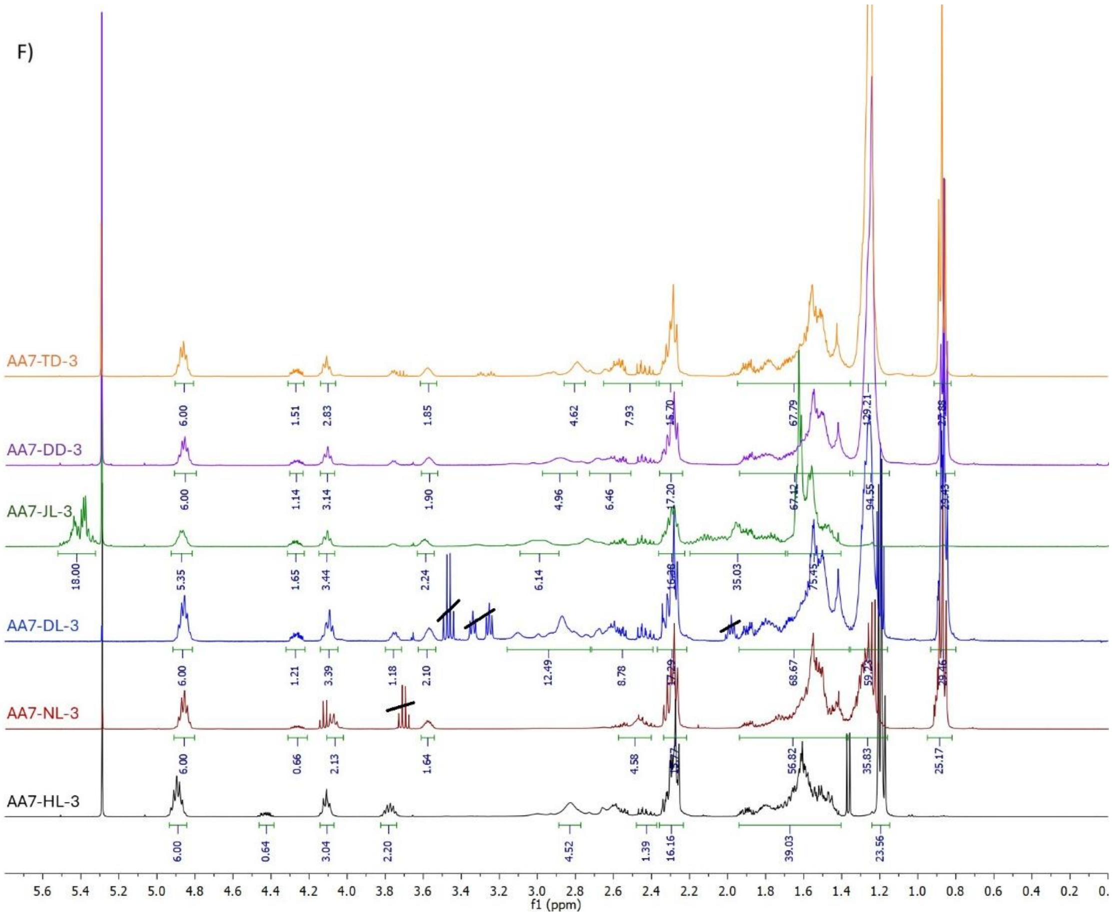

Figure S2. GPC chromatograms for the APEs library with different alkyl side chain composition.

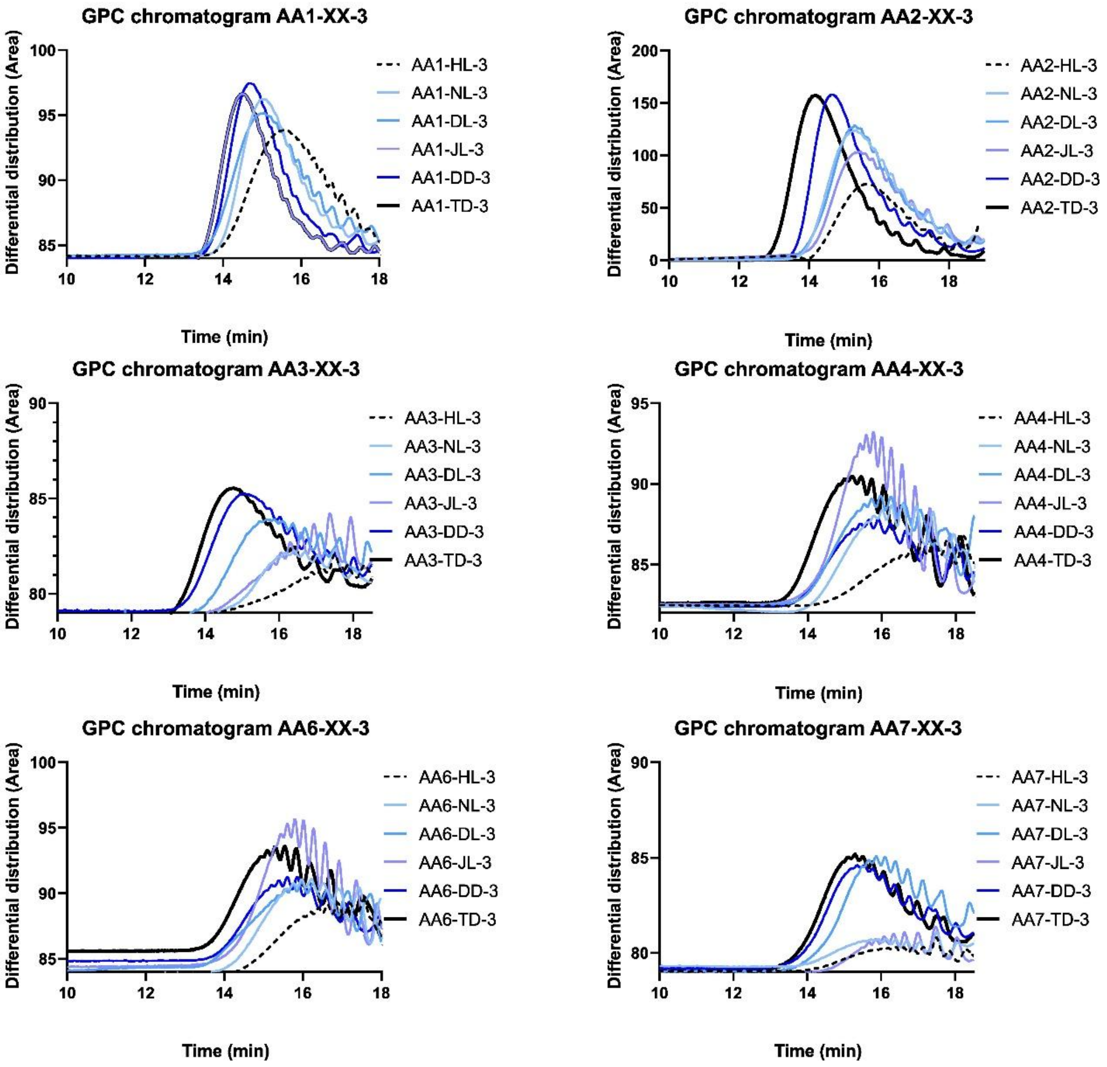

Table S1. 1H-NMR Characterization of the synthesized amino-polyesters (APEs).

APE	Mn_{theo}	Conversion (%)	q_{NMR}
AA1-HL-3	1606.11	80.9	2.7
AA1-NL-3	2110.95	85.2	2.5
AA1-DL-3	2279.31	81.2	3.3
AA1-JL-3	2255.31	78.8	2.9
AA1-DD-3	2615.91	80.9	2.9
AA1-TD-3	2952.63	86.6	2.7
AA2-HL-3	1747.37	94	2.4
AA2-NL-3	2252.21	86.3	2
AA2-DL-3	2420.57	84.1	1.9
AA2-JL-3	2396.33	85.1	2.1
AA2-DD-3	2757.17	88.7	2
AA2-TD-3	3093.89	82	3.1
AA3-HL-3	1389.91	90	2.8
AA3-NL-3	1768.54	84.3	2.7
AA3-DL-3	1894.81	76.8	2
AA3-JL-3	1876.63	85.8	2.5
AA3-DD-3	2147.26	78	2.1
AA3-TD-3	2399.8	84.8	2.4
AA4-HL-3	889.21	92.7	2.4
AA4-NL-3	1141.63	87.5	2.1
AA4-DL-3	1225.81	82.5	2.7
AA4-JL-3	1213.69	82.3	1.7
AA4-DD-3	1394.11	85	1.9
AA4-TD-3	1562.47	87.2	2
AA6-HL-3	889.21	92.5	2.2
AA6-NL-3	1141.63	91.8	2.8

AA6-DL-3	1225.81	94	3.7
AA6-JL-3	1213.69	-	-
AA6-DD-3	1394.11	92.3	2.5
AA6-TD-3	1562.47	84	2.2
AA7-HL-3	1330.8	92.5	3.3
AA7-NL-3	1709.43	86.5	2.5
AA7-DL-3	1835.7	87	2.4
AA7-JL-3	1835.7	82.1	2.2
AA7-DD-3	2088.15	83.6	2.7
AA7-TD-3	2340.69	83.2	2.8

Characterization by ^{1}H-NMR of APEs library. Mn_{theo}: theoretical molecular weight (g/mol), q_{NMR}: degree of polymerization (calculated as described below).

Conversion of lactones is evaluated according to (D, G, g, H described in Figure S1):

$$Conversion\ (\%) = \frac{G + D}{\mathrm{G + D + g}} * 100$$

Degree of polymerization (q_{NMR}) is evaluated according to:

$$q_{NMR} = \frac{\mathrm{G + D}}{H}$$

Characterization and calculations are performed as previously reported by Kowalski et al.[28]

Table S2. Characterization of selected APEs and APE-LNPs.

APE	Mn[a)] [kDa]	Đ[a)] [-]	Diameter[c)] [nm]	PDI[c)] [-]	ζ[c)] [mV]	mRNA EE[d)] [%]	pK_a[e)]
AA1-HL-3	1.85 ± 0.09	1.28 ± 0.03	236 ± 59	0.110 ± 0.02	-26.5 ± 11.3	14.2 ± 8.5	3.3 ± 0.1
AA1-NL-3	2.34 ± 0.13	1.24 ± 0.04	139 ± 7	0.121 ± 0.08	-23.2 ±14.5	40.2 ± 6.8	3.4 ± 0.1
AA1-DL-3	2.40 ± 0.01	1.27 ± 0.00	129 ± 3	0.072 ± 0.03	-22.9 ± 2.7	51.8 ± 5.1	3.3 ± 0.0
AA1-JL-3	2.03 ± 0.09	1.37 ± 0.03	133 ± 14	0.065 ± 0.01	-32.8 ± 9.1	23.0 ± 11.9	3.4 ± 0.1
AA1-DD-3	2.87 ± 0.02	1.25 ± 0.03	99 ± 4	0.128 ± 0.01	-12.3 ± 8.0	74.6 ± 8.0	3.5 ± 0.2
AA1-TD-3	3.44 ± 0.59	1.23 ± 0.08	77 ± 8	0.133 ± 0.02	-12.2 ± 7.3	81.2 ± 4.0	3.6 ± 0.1
AA3-HL-3	1.12 ± 0.24	1.38 ± 0.09	238 ± 30	0.380 ±0.29	-5.7 ± 0.6	85.7 ± 6.5	5.4 ± 0
AA3-NL-3	1.37 ± 0.03	1.23 ± 0.01	70 ± 3	0.138 ± 0.06	-0.1 ± 0.9	97.1 ± 0.8	5.2 ± 0.1
AA3-DL-3	1.43 ± 0.29	1.39 ± 0.04	70 ± 14	0.197 ± 0.07	2.4 ± 1.1	98.1 ± 0.6	5.4 ± 0.2
AA3-JL-3	1.10 ± 0.13	1.27 ± 0.02	76 ± 4	0.101 ± 0.01	-1.5 ± 2.1	90.3 ± 6.4	5.2 ± 0.1
AA3-DD-3	1.51 ± 0.08	1.29 ± 0.03	66 ± 9	0.210 ± 0.02	2.0 ± 1.7	98.6 ± 0.9	5.0 ± 0.1
AA3-TD-3	2.07 ± 0.14	1.24 ± 0.03	85 ± 22	0.241 ± 0.00	2.6 ± 2.5	98.6 ± 0.8	5.1 ± 0.1
AA4-HL-3	1.14 ± 0.13	1.33 ± 0.02	281 ± 17	0.280 ± 0.17	-12.9 ± 8.8	45.3 ± 9.5	4.6 ± 0.6
AA4-NL-3	1.44 ± 0.07	1.30 ± 0.05	78 ± 0	0.188 ± 0.00	-4.0 ± 4.9	87.1 ± 10.8	4.6 ± 0.4
AA4-DL-3	1.55 ± 0.29	1.36 ± 0.04	70 ± 16	0.197 ± 0.04	-2.6 ± 1.7	84.6 ± 14.9	4.2 ± 0.1
AA4-JL-3	1.80 ± 0.02	1.27 ± 0.01	86 ± 8	0.102 ± 0.02	-8.1 ± 10.2	50.0 ± 9.4	4.2 ± 0.1

AA4-DD-3	1.60 ± 0.08	1.35 ± 0.07	67 ± 18	0.200 ± 0.07	-3.1 ± 2.8	90.4 ± 7.9	4.1 ± 0.3
AA4-TD-3	1.91 ± 0.00	1.36 ± 0.00	65 ± 6	0.212 ± 0.01	-1.4 ± 0.8	92.0 ± 6.3	4.2 ± 0.2
AA6-HL-3	1.20 ± 0.05	1.29 ± 0.02	174 ± 33	0.177 ± 0.01	-20,7 ± 5.1	40.1 ± 8.3	4.4 ± 0.1
AA6-NL-3	1.44 ± 0.05	1.33 ± 0.00	119 ± 18	0.158 ± 0.05	-17.0 ± 5.7	81.5 ± 14.1	4.3 ± 0.1
AA6-DL-3	1.61 ± 0.04	1.35 ± 0.03	109 ± 19	0.184 ± 0.03	-10.5 ± 2.0	80.1 ± 17.0	4.1 ± 0.1
AA6-JL-3	1.75 ± 0.03	1.26 ± 0.00	106 ± 21	0.220 ± 0.03	-20.9 ± 9.7	46.1 ± 0.2	-
AA6-DD-3	2.08 ± 0.03	1.23 ± 0.02	83 ± 21	0.217 ± 0.03	-2.3 ± 0.7	94.7 ± 0.7	4.2 ± 0.1
AA6-TD-3	2.09 ± 0.15	1.31 ± 0.07	89 ± 21	0.234 ± 0.05	-7.6 ± 3.4	96.1 ± 1.3	4.2 ± 0.1
AA7-HL-3	1.36 ± 0.03	1.36 ± 0.02	229 ± 40	0.343 ± 0.24	0.5 ± 2.4	87.6 ± 10.0	4.4 ± 0.4
AA7-NL-3	1.70 ± 0.01	1.33 ± 0.10	67 ± 7	0.117 ± 0.06	4.7 ± 4.8	95.6 ± 3.1	4.8 ± 0.8
AA7-DL-3	1.78 ± 0.08	1.44 ± 0.02	65 ± 0	0.163 ± 0.02	4.6 ± 2.8	96.1 ± 3.0	4.8 ± 0.5
AA7-JL-3	1.33 ± 0.06	1.29 ± 0.00	68 ± 3	0.078 ± 0.00	4.4 ± 5.2	89.9 ± 7.5	5.6 ± 1.1
AA7-DD-3	1.76 ± 0.21	1.46 ± 0.02	59 ± 2	0.153 ± 0.05	3.7 ± 4.7	93.7 ± 6.2	4.9 ± 0.1
AA7-TD-3	1.62 ± 0.03	1.44 ± 0.04	65 ± 5	0.178 ± 0.02	5.4 ± 5.4	94.2 ± 5.0	4.1 ± 0.1

Data are represented as mean ± SD from two independently synthesized batches of polymer and corresponding APE-LNPs (n = 2); [a)]Characterized by Gel-permeation chromatography; [b)]Characterized by dynamic light scattering; [c)]Characterized by Quant-iT RiboGreen; [d)]Characterized by TNS assay; Mn: number average molecular weight, Đ: dispersity, PDI: polydispersity index, ζ: zeta potential, EE: encapsulation efficacy.

Figure S3. Glass transition (T_g) of selected polymers. DSC curve for polymers with amino-alcohols AA1, AA3 and AA4 with 3 monomer repetitions (HL, JL, NL, DL and DD).

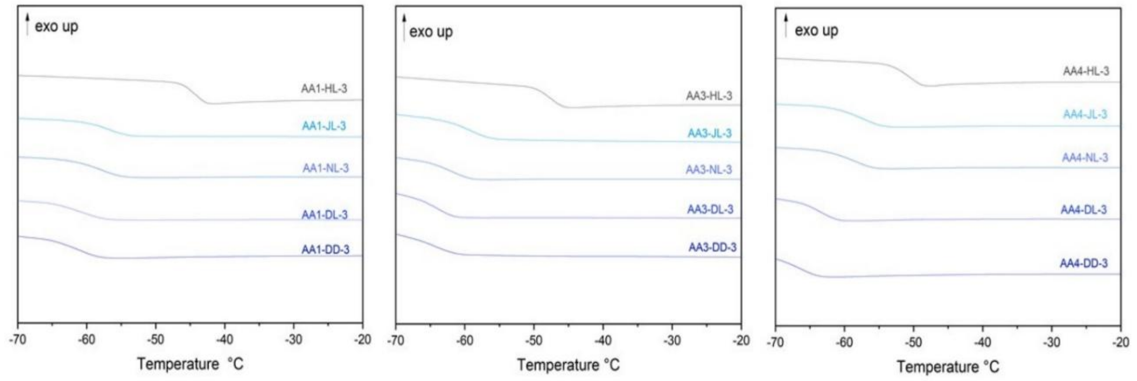

Table S3. MW of APEs with 10 lactone monomers

APE	Mn_{theo}	$Mn^{a)}$ [Da]	$Đ^{a)}$ [-]
AA1-DD-10	8168.31	6761	1.19
AA4-DD-10	4170.31	3404	1.45

Characterization of AA1-DD-10 and AA4-DD-10 by [a)]Characterized by Gel-permeation chromatography. Mn_{theo}: theoretical molecular weight (g/mol), Mn: number average molecular weight, Đ: dispersity.

Figure S4. APE-LNPs characterization and *in vitro* evaluation in HeLa cells.

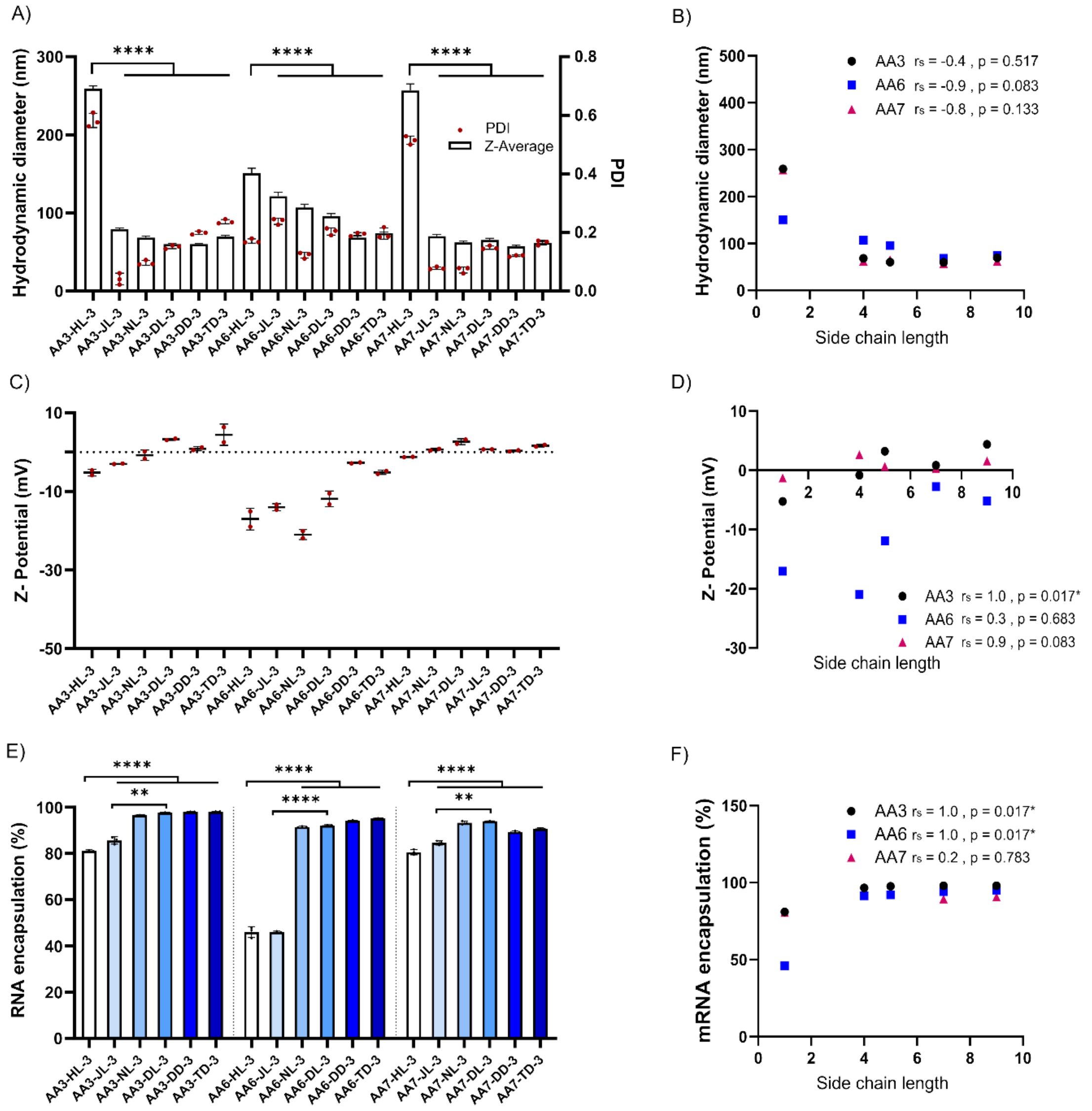

A) APE-LNPs hydrodynamic diameter (nm) and polydispersity index (PDI) for polymers AA3, AA6 and AA7 with different monomer side chain length. Data are presented as mean ± SD; n = 3. **** $p < 0.000\ 1$. C) APE-LNPs surface charge. Data are presented as mean ± SD; n = 2. E) APE-LNPs mRNA encapsulation efficacy determined with Ribogreen assay. Data are presented as mean ± SD; n = 3. ** $p < 0.001$, **** $p < 0.000\ 1$. Spearman correlation coefficient between polymer side chain length and B) hydrodynamic diameter, D) surface charge, F) mRNA encapsulation.

Figure S5. Additional Cryo-TEM images of AA2-HL-3 and AA2-NL-3 nanoparticles.

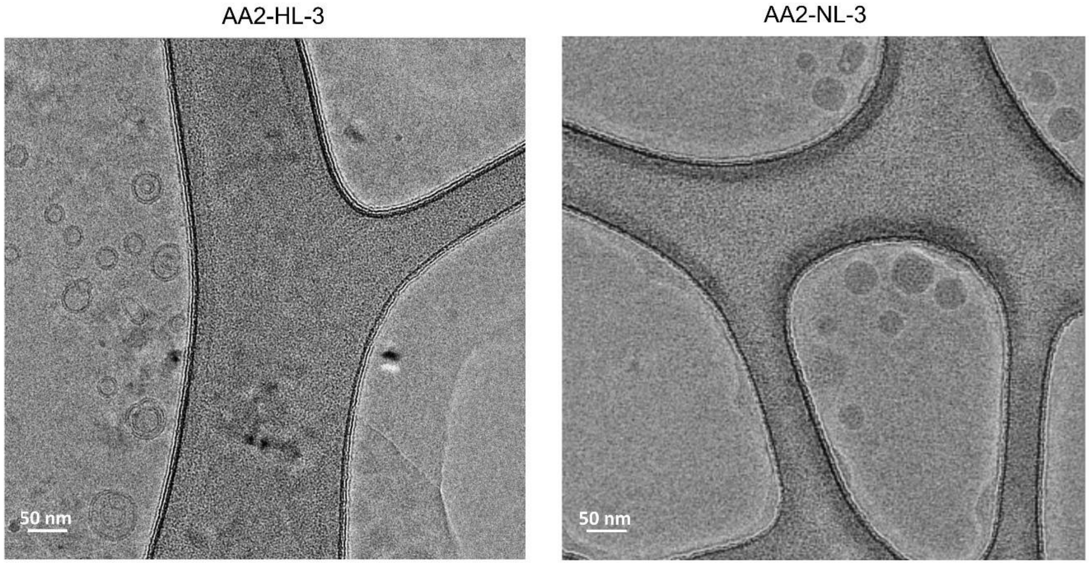

Scale bar represents 50 nm.

Figure S6. Changes in hydrodynamic diameter (nm) and polydispersity index (PDI) of selected APE-LNPs were determined by dynamic light scattering (DLS). Measurements were performed every 7 days after formulation for the period of 28 days. Data are presented as mean ± SD; n = 3.

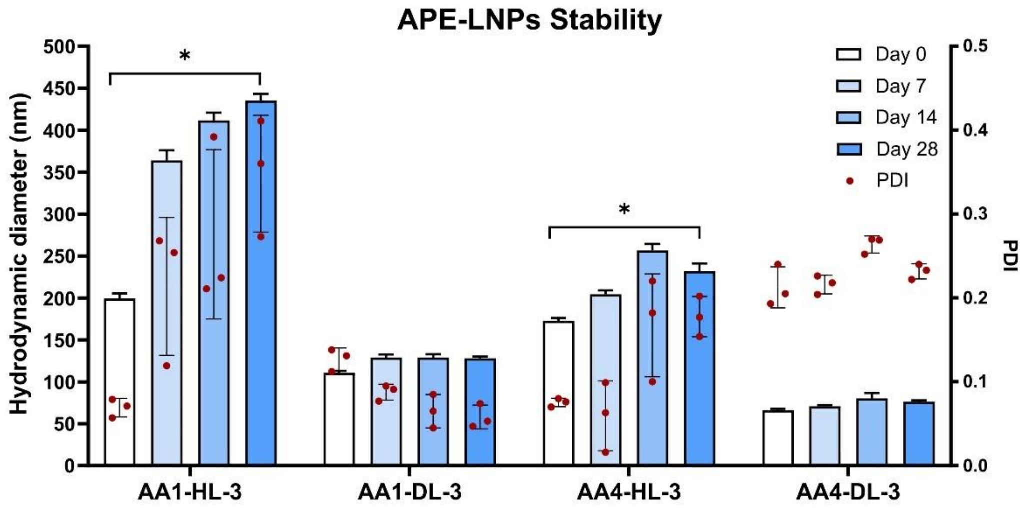

Figure S7. *In vitro* evaluation of mRNA delivery capacity by APE-LNPs containing FLuc mRNA. Cells were transfected for 24 h with 50 ng of FLuc mRNA.

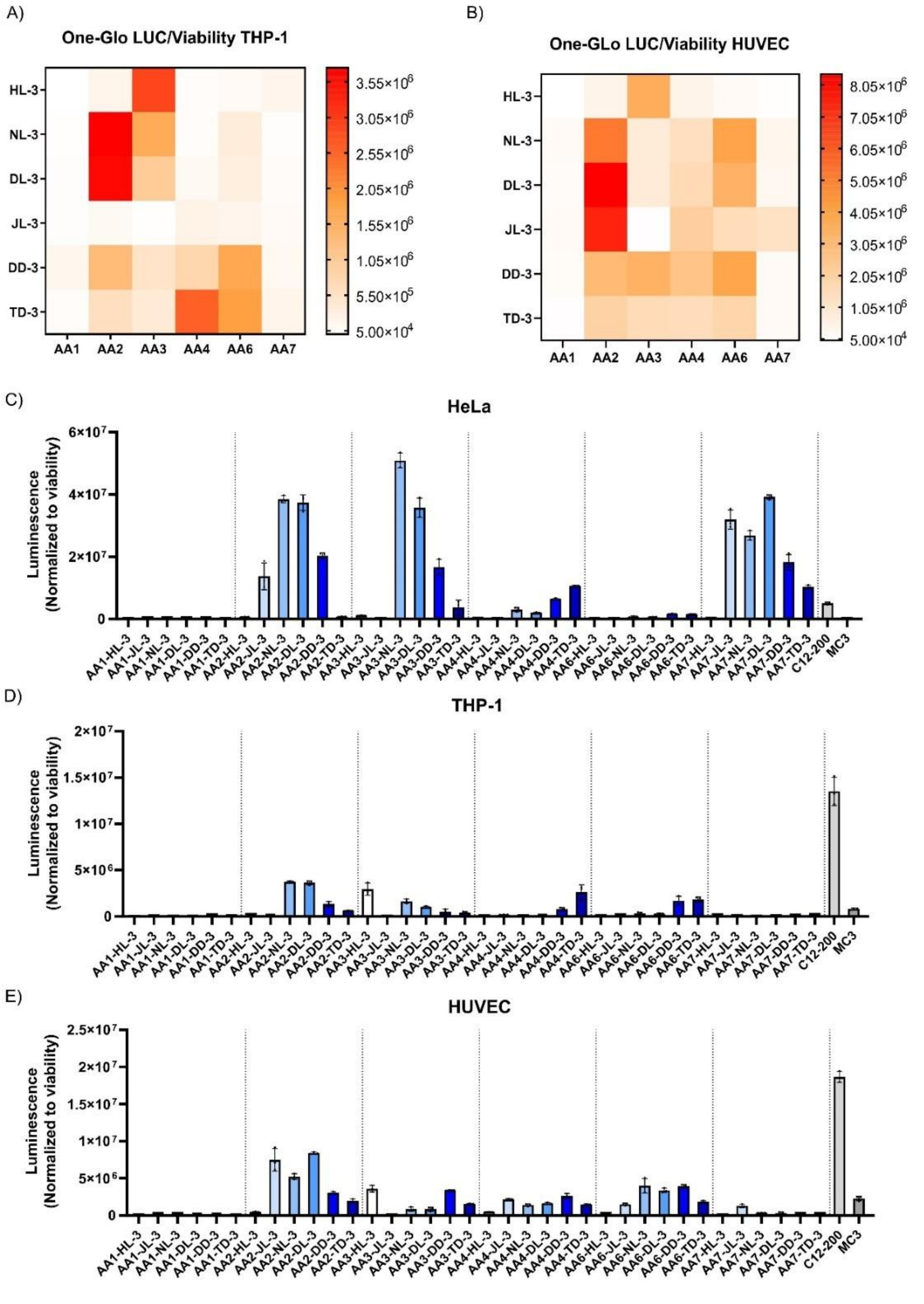

Heat map representation of luminescence (normalized to viability) of APE-LNPs delivery efficacy for A) THP-1 and B) HUVEC. Data are presented as mean relative luminescence (normalized to viability); n = 3. Luminescence of APE-LNPs containing FLuc mRNA normalized to viability in C) HeLa, D) THP-1 and E) HUVEC, including C12-200 as reference. Data are presented as mean ± SD relative luminescence (normalized to viability); n = 3.

Figure S8. Cell viability 24 h after transfection in A) HeLa, B) THP-1 and C) HUVEC.

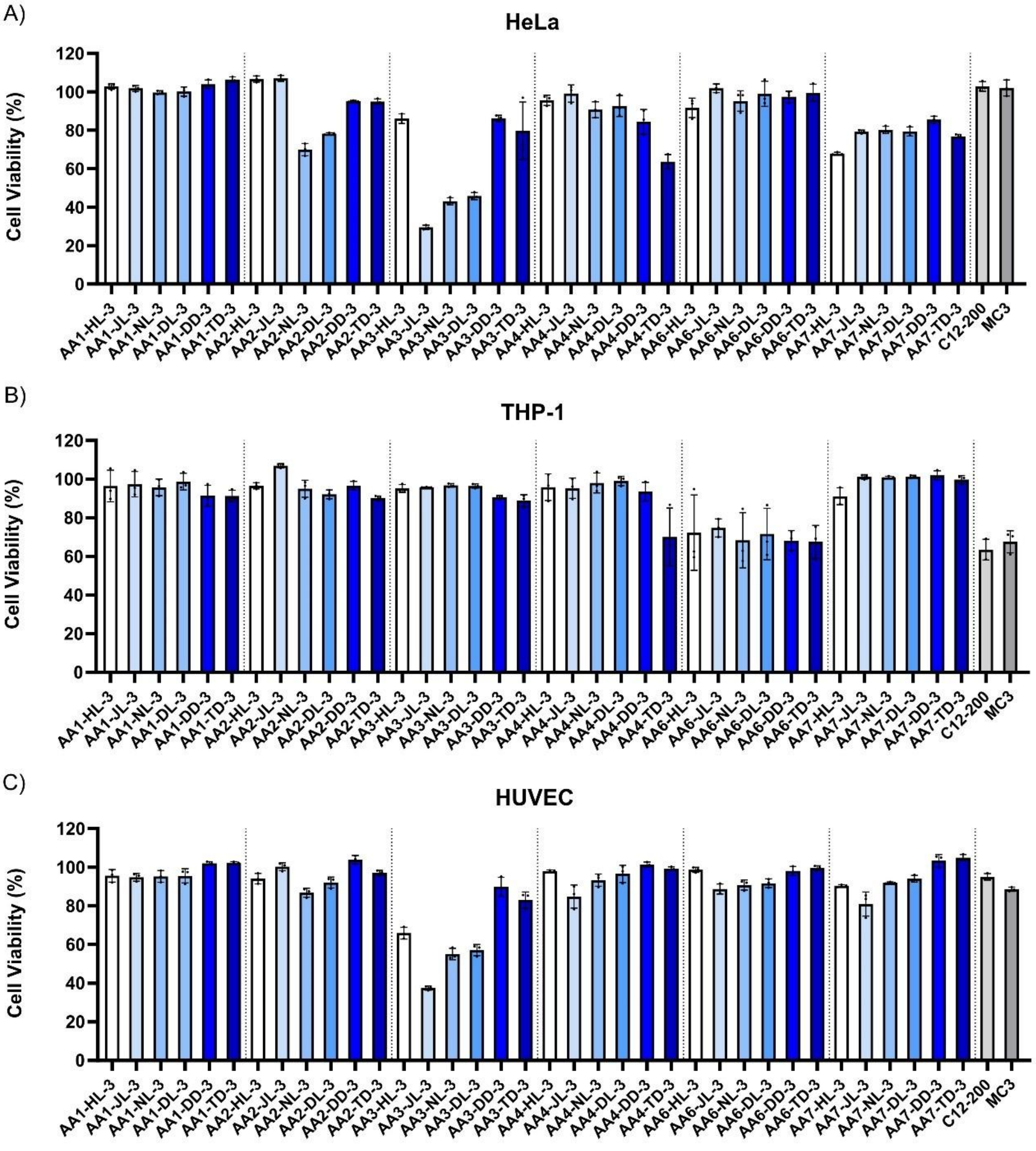

Data are presented as mean ± SD; n = 3.

Figure S9: A) pK_a of APE-LNPs determined with TNS assay. Data are presented as mean; n = 2. X denotates that pK_a could not be determined. B) Representative pK_a curves for selectes polymers. Data are presented as mean; n = 2. Ionization score at C) pH 5 and D) pH 7 of selected polymers determined with TNS assay. Data are presented as mean; n = 2.

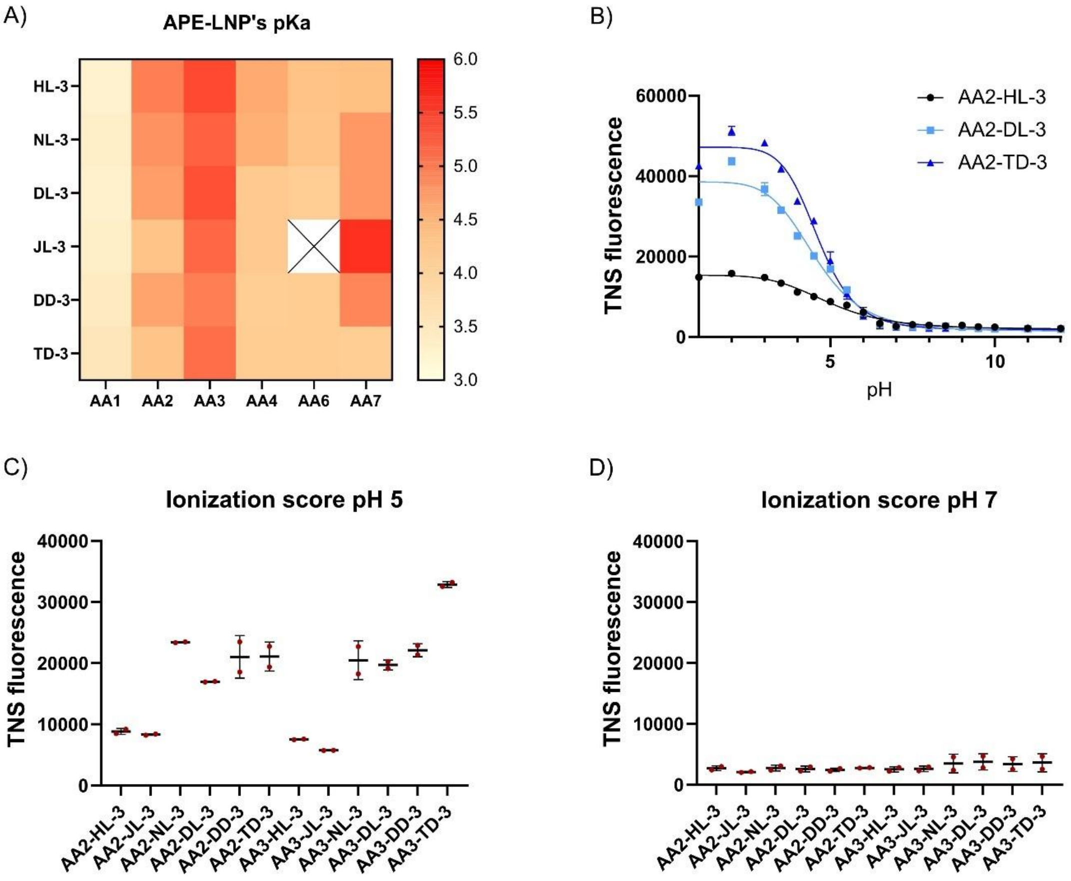

Table S4. Formulations to study the role of helper lipids.

Formulation	AA2-NL-3	DOPE	Cholesterol	C14-PEG2000
APE-LNP 4C	50 (50.0)	25 (25.0)	23.5 (23.5)	1.5 (1.5)
(-) Cholesterol	50 (65.4)	25 (32.7)	-	1.5 (2.0)
(-) DOPE	50 (66.7)	-	23.5 (31.3)	1.5 (2.0)
(-) DOPE (-) Cholesterol	50 (97.1)	-	-	1.5 (2.9)

Molar ratio (Mol %)

Figure S10. Characterization of AA2-NL-3 APE-LNPs with/without helper lipids.

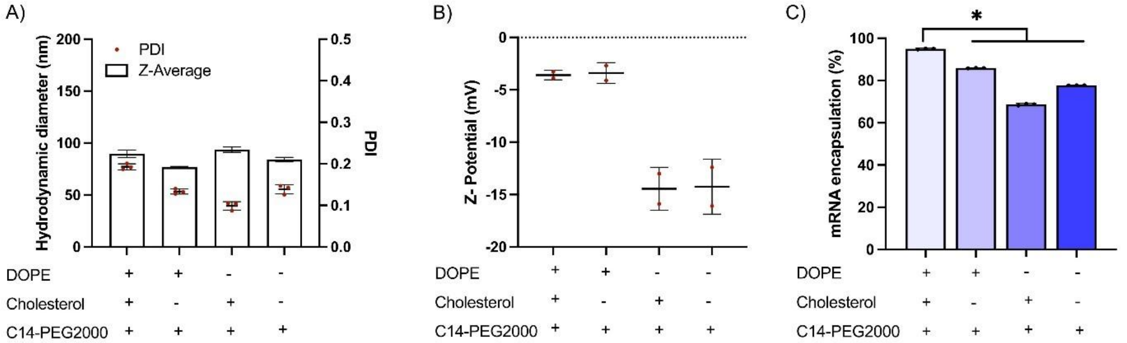

A) Hydrodynamic diameter (nm) and polydispersity index (PDI) of APE-LNPs are presented as mean ± SD; n = 3. B) APE-LNPs surface charge determined with Z-potential. Data presented as mean ± SD; n = 2. C) mRNA encapsulation efficacy of APE-LNPs determined with Ribogreen assay. Data are presented as mean ± SD; n = 3. * p < 0.05, *** p <0.005.

Figure S11. Investigation of the role of helper lipids in AA2-NL-3 APE-LNPs in HeLa cells.

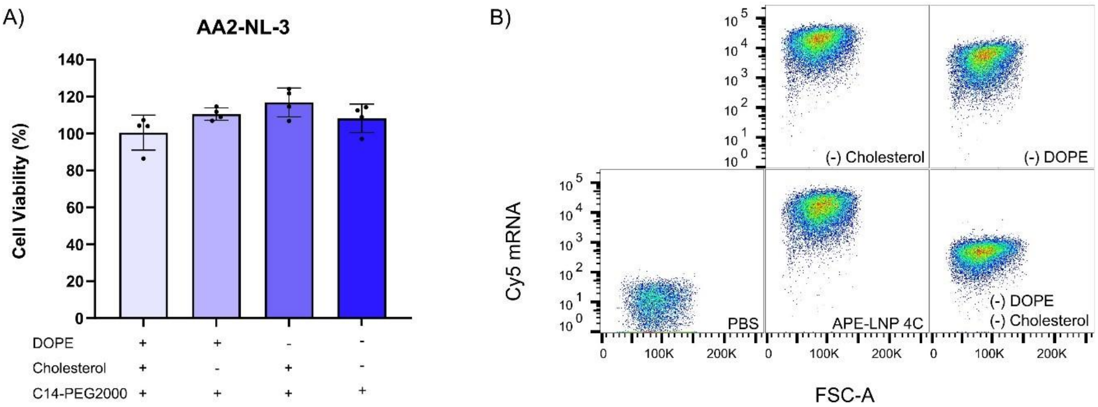

A) Cell viability of HeLa cells 24 h after transfection with 50 ng of APE-LNPs containing FLuc mRNA. Data are presented as mean ± SD; *n* = 3. B) Dot plots of $Cy5^{+}$ cell populations treated with PBS or APE-LNPs (4-component (4C), (-) DOPE and/or (-) cholesterol) containing Cy5 mRNA.

Figure S12. Investigation of the role of helper lipids in AA4-NL-3 APE-LNPs containing Cy5-labeled FLuc mRNA in HeLa cells.

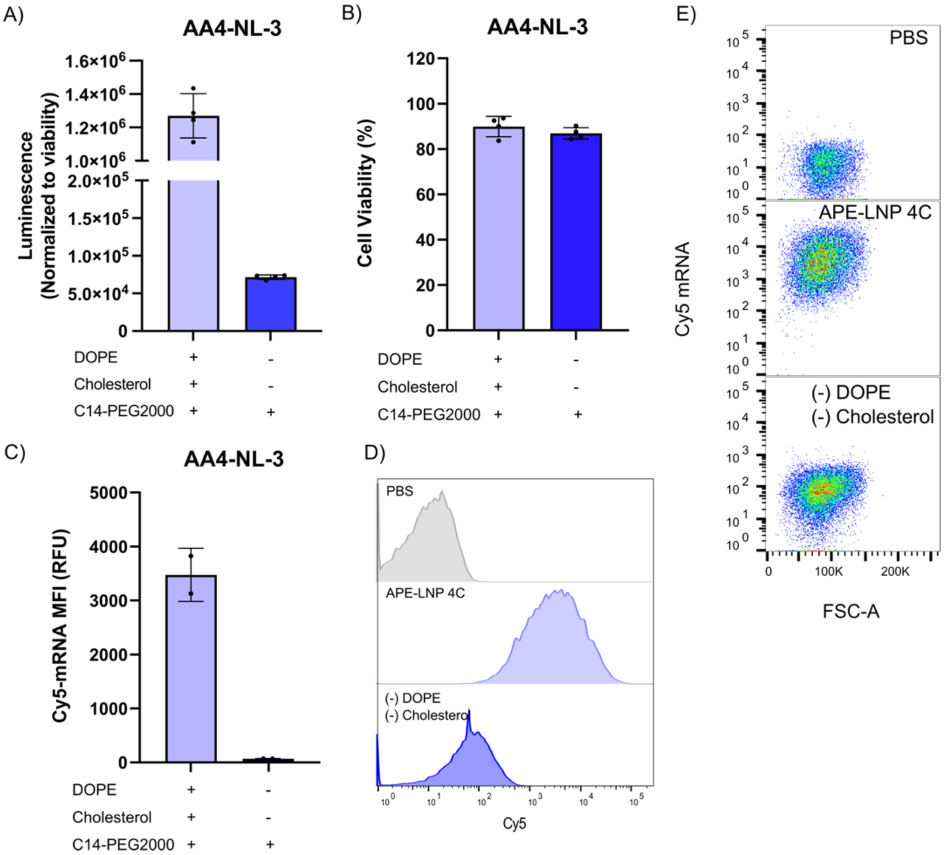

A) mRNA delivery efficacy of AA4-NL-3 APE-LNPs with or without helper lipids. Cells were transfected for 24 h with 50 ng of FLuc mRNA. Data are presented as mean relative luminescence (normalized to viability) ± SD; n = 3. B) Cell viability in HeLa cells 24 h after transfection. C) Flow cytometry analysis of the uptake of AA4-NL-3 APE-LNPs with/without helper lipids containing Cy5 mRNA, 24 h after transfection. Data are presented as mean ± SD; n = 3. Data are presented as mean ± SD; n = 3. D) Representative histograms and E) Dot plots of $Cy5^{+}$ cell populations treated with PBS or APE-LNPs (4-component (4C), (-) DOPE and (-) cholesterol) containing Cy5 mRNA.

Figure S13. In vivo evaluation of APEs. C57BL/6 mice were injected via tail vein with 0.5 mg/kg of APE-LNPs containing mRNA encoding Firefly luciferase (FLuc) mRNA and imaged by IVIS after 6h.

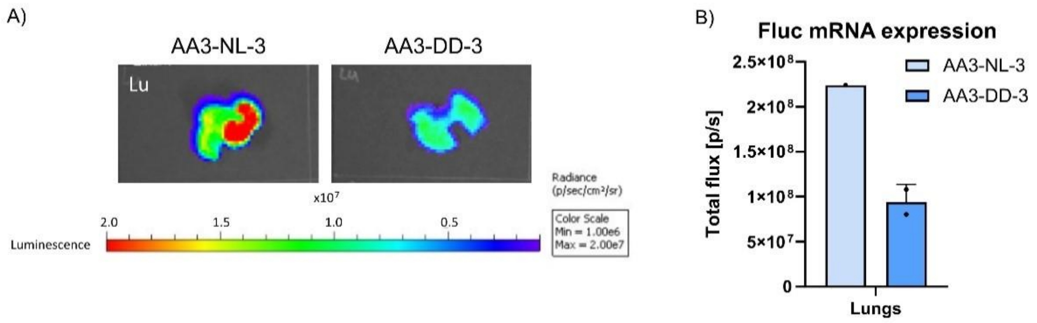

A) Representative images of FLuc mRNA expression within the lungs (Lu). B) Quantification of FLuc mRNA expression in the lungs. Data are presented as mean ± SD; n = 2.

Figure S14. In vivo evaluation of APE-LNPs biodistribution. C57BL/6 mice were injected via tail vein with 0.5 mg/kg of DiR dye labeled APE-LNPs and imaged by IVIS after 6h

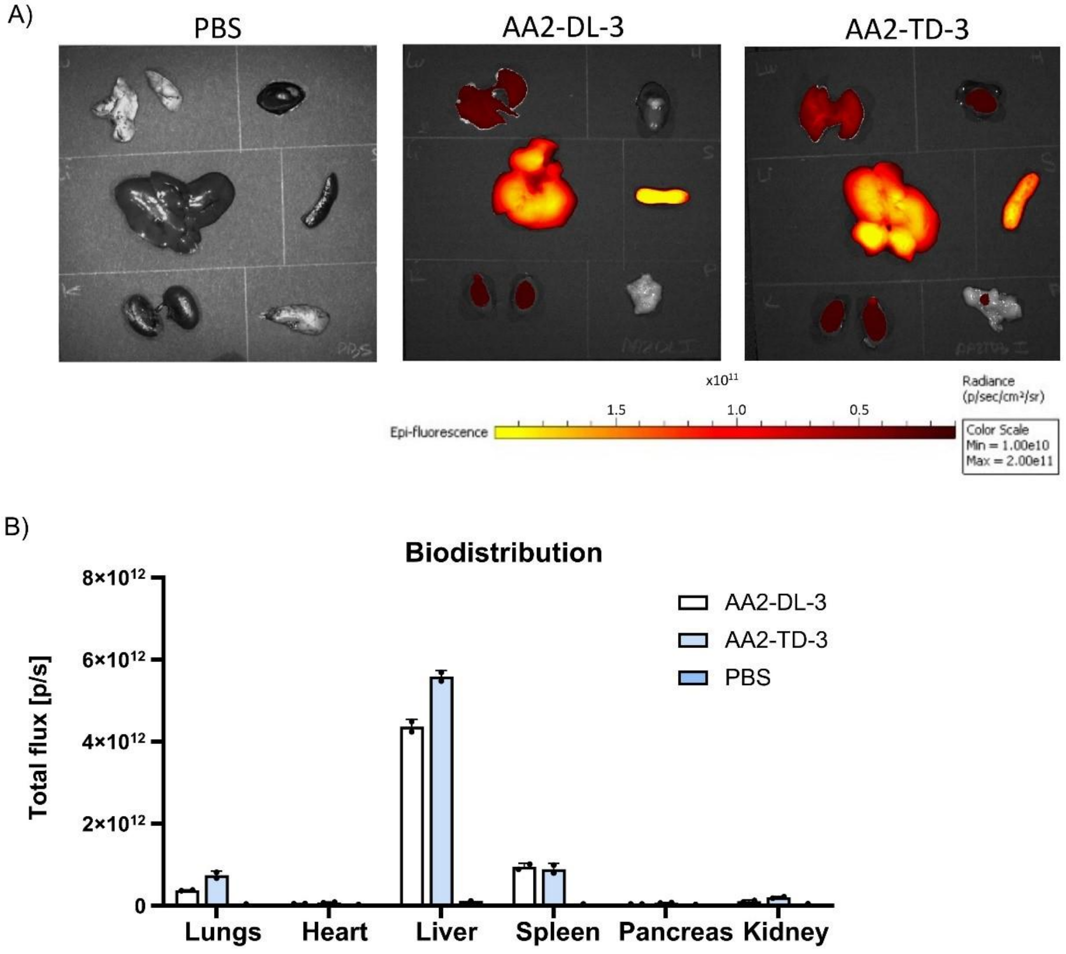

.

A) Representative images of DiR dye fluorescence expression within the tissues; Lu: lungs, H: heart, Li: liver, S: spleen, K: kidney, P: pancreas. B) Quantification of DiR fluorescence expression in selected tissues. Data are presented as mean ± SD; n = 2.

Figure S15. APE-LNPs for in vivo screening characterization.

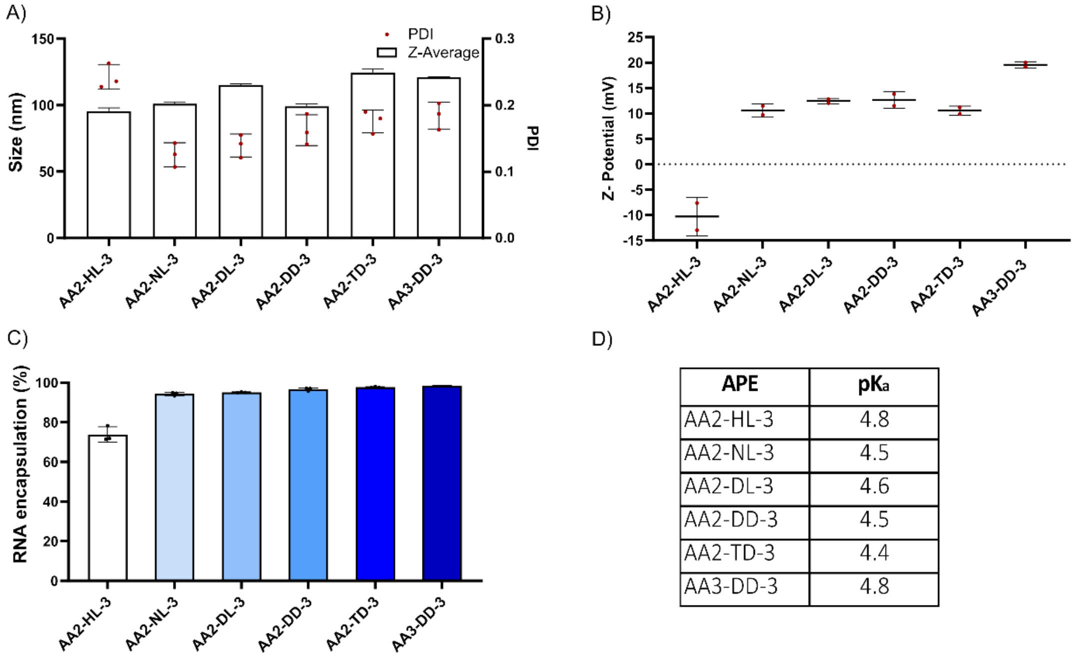

APE	pKa
AA2-HL-3	4.8
AA2-NL-3	4.5
AA2-DL-3	4.6
AA2-DD-3	4.5
AA2-TD-3	4.4
AA3-DD-3	4.8

A) APE-LNPs hydrodynamic diameter (nm) and polydispersity index (PDI). Data are presented as mean ± SD; n = 3. B) APE-LNPs surface charge determined with Z-potential. Data are presented as mean ± SD; n = 2. C) APE-LNPs mRNA encapsulation efficacy determined with Ribogreen. Data are presented as mean ± SD; n = 3. D) pK_a of APE-LNPs determined with TNS assay.

Table S5. Amino-alcohols yield

Amino-alcohol	Yield (%)
AA2	76
AA3	82
AA4	87
AA6	68
AA7	95

Characterization of amino-alcohols

Synthesized amino-alcohols were characterized by NMR and ESI.

Amino-alcohol AA2: ^{1}H NMR (400 MHz, CDCl$_3$) δ 3.65 (t, 8H, *J* = 5.7 Hz, H1, 2.54 (t, 8H, *J* = 6.6 Hz, H3), 2.40 (t, 4H, *J* = 7.1 Hz, H6), 2.32 (t, 4H, *J* = 7.1 Hz, H4), 2.16 (s, 3H, H7), 1.73-1.62(m, 12H, H2, H5). ^{13}C NMR (MHz, CDCl$_3$) δ 62.39 (C1), 55.60 (C6), 53.10 (C3), 52.25 (C4), 42.49 (C7), 28.87 (C2), 24.67 (C5). ESI $C_{19}H_{43}N_3O_4$ (M + H$^+$) calcd. 377.6 obsd. 378.4

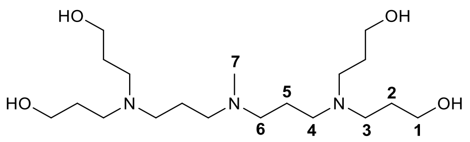

Amino-alcohol AA3: ^{1}H NMR (400 MHz, CDCl$_3$) δ 3.75 (t, 6H, *J* = 5.2 Hz, H1), 2.62-2.54 (m, 12H, H5, H6), 2.48 (t, 6H, *J* = 7.7 Hz, H3), 2.25 (s, 9H, H4), 1.67 (quin, 6H, *J* = 5.2, 7.7 Hz, H2). ^{13}C NMR (MHz, CDCl$_3$) δ 63.66 (C1), 57.60 (C5), 55.60 (C3), 52.63 (C6), 42.66 (C4), 28.20 (C2). ESI $C_{18}H_{42}N_4O_3$ (M + H$^+$) calcd. 362.6 obsd. 363.3.

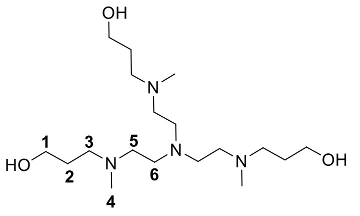

Amino-alcohol AA4: ^{1}H NMR (400 MHz, CDCl$_3$) δ 3.72 (t, 4H, *J* = 5.5 Hz, H1), 2.57 (t, 4H, *J* = 6.1 Hz, H3), 2.52 (t, 2H, *J* = 5.9 Hz, H5), 2.44 (t, 2H, *J* = 5.9 Hz, H4), 2.23 (s, 6H, H6), 1.68 (quin, 4H, *J* = 5.5, 6.1 Hz, H2). ^{13}C NMR (MHz, CDCl$_3$) δ 61.72 (C1), 57.51 (C5), 52.43 (C3), 51.19 (C4), 45.34 (C6), 29.08 (C2). ESI $C_{10}H_{24}N_2O_2$ (M + H$^+$) calcd. 204.3 obsd. 205.4.

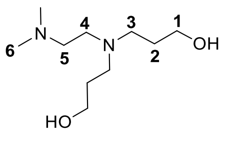

Amino-alcohol AA6: ^{1}H NMR (400 MHz, CDCl$_3$) δ 5.04 (s, 2H, OH), 3.75 (t, 4H, *J* = 5.3 Hz, H1), 2.56 (t, 4H, *J* = 5.8 Hz, H3), 2.49 (s, 4H, H5), 2.25 (s, 6H, H4), 1.68 (quin, 4H, *J* = 5.3, 5.8 Hz H2). ^{13}C NMR (MHz, CDCl$_3$) δ 63.19 (C1), 57.05 (C5), 55.69 (C3), 42.36 (C4), 28.40 (C2). ESI $C_{10}H_{24}N_2O_2$ (M + H$^+$) calcd. 204.3 obsd. 205.4.

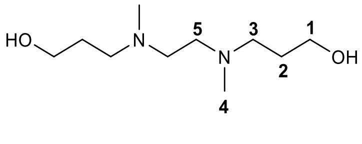

Amino-alcohol AA7: ^{1}H NMR (400 MHz, CDCl$_3$) δ 3.73 (t, 6H, *J* = 5.4 Hz H1), 2.71-2.63 (m, 18H, H3, H4), 1.62 (quin, 6H, *J* = 5.4, 5.7 Hz, H2). ^{13}C NMR (MHz, CDCl$_3$) δ 62.70 (C1), 60.69 (C3), 54.16 (C4), 27.93 (C2). ESI $C_{19}H_{43}N_3O_4$ (M + H$^+$) calcd. 303.5 obsd. 304.2.

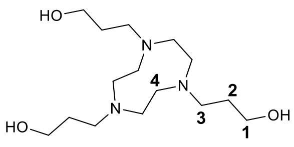
HO
HO
N
N
N
4
2
3
1
OH
